# MEK inhibition ameliorates social behavior phenotypes in a Spred1 knockout mouse model for RASopathy disorders

**DOI:** 10.1186/s13229-021-00458-2

**Published:** 2021-07-26

**Authors:** Sarah C. Borrie, Ellen Plasschaert, Zsuzsanna Callaerts-Vegh, Akihiko Yoshimura, Rudi D’Hooge, Ype Elgersma, Steven A. Kushner, Eric Legius, Hilde Brems

**Affiliations:** 1 Department of Human Genetics, KU Leuven, O&N1 Herestraat 49, Box 607, 3000 Leuven, Belgium; 2 Laboratory for Biological Psychology, KU Leuven, Leuven, Belgium; 3 Department of Microbiology and Immunology, Keio University School of Medicine, Tokyo, Japan; 4 ENCORE Expertise Center for Neurodevelopmental Disorders, Erasmus MC University Medical Center, Rotterdam, The Netherlands; 5 Department of Neuroscience, Erasmus MC University Medical Center, Rotterdam, The Netherlands; 6 Department of Psychiatry, Erasmus MC University Medical Center, Rotterdam, The Netherlands

**Keywords:** RASopathy, Autism spectrum disorder, Spred1, Neurofibromatosis type 1, Social dominance, Ultrasonic vocalization

## Abstract

**Background:**

RASopathies are a group of disorders that result from mutations in genes coding for proteins involved in regulating the Ras-MAPK signaling pathway, and have an increased incidence of autism spectrum disorder (ASD). Legius syndrome is a rare RASopathy caused by loss-of-function mutations in the *SPRED1* gene. The patient phenotype is similar to, but milder than, Neurofibromatosis type 1—another RASopathy caused by loss-of-function mutations in the *NF1* gene. RASopathies exhibit increased activation of Ras-MAPK signaling and commonly manifest with cognitive impairments and ASD. Here, we investigated if a *Spred1-/-* mouse model for Legius syndrome recapitulates ASD-like symptoms, and whether targeting the Ras-MAPK pathway has therapeutic potential in this RASopathy mouse model.

**Methods:**

We investigated social and communicative behaviors in *Spred1-/-* mice and probed therapeutic mechanisms underlying the observed behavioral phenotypes by pharmacological targeting of the Ras-MAPK pathway with the MEK inhibitor PD325901.

**Results:**

*Spred1-/-* mice have robust increases in social dominance in the automated tube test and reduced adult ultrasonic vocalizations during social communication. Neonatal ultrasonic vocalization was also altered, with significant differences in spectral properties. *Spred1-/-* mice also exhibit impaired nesting behavior. Acute MEK inhibitor treatment in adulthood with PD325901 reversed the enhanced social dominance in *Spred1-/-* mice to normal levels, and improved nesting behavior in adult *Spred1-/-* mice.

**Limitations:**

This study used an acute treatment protocol to administer the drug. It is not known what the effects of longer-term treatment would be on behavior. Further studies titrating the lowest dose of this drug that is required to alter *Spred1-/-* social behavior are still required. Finally, our findings are in a homozygous mouse model, whereas patients carry heterozygous mutations. These factors should be considered before any translational conclusions are drawn.

**Conclusions:**

These results demonstrate for the first time that social behavior phenotypes in a mouse model for RASopathies (*Spred1-/-*) can be acutely reversed. This highlights a key role for Ras-MAPK dysregulation in mediating social behavior phenotypes in mouse models for ASD, suggesting that proper regulation of Ras-MAPK signaling is important for social behavior.

**Supplementary Information:**

The online version contains supplementary material available at 10.1186/s13229-021-00458-2.

## Background

Autism Spectrum Disorder (ASD) is a genetically heterogeneous disorder of neurodevelopment. Syndromic ASD, in which rare genetic syndromes have an increased penetrance for ASD, accounts for ~ 5% of overall ASD incidence [1]. Diagnosis of ASD requires two clinical criteria: (1) deficits in social interaction and social communication, and (2) restricted, repetitive patterns of behavior, which can include hyper- or hypoactivity to sensory input [2]. RASopathy disorders stem from mutations in genes encoding regulators of Ras-mitogen-activated protein kinase (Ras-MAPK) signaling, and patients exhibit heightened incidence of ASD [3–8]. RASopathies also result in overlapping phenotypes in cardiac, craniofacial and dermatological domains, as well as increased tumor risk [9]. The RASopathy Neurofibromatosis type 1 (NF1) is a major syndromic form of ASD, resulting from mutations in the *NF1* gene encoding for neurofibromin, a negative regulator of Ras. Social problems among children and adults with NF1 are well documented [10–12], and it is estimated that 10–25% of patients have ASD [5, 13, 14]. Incidence of ASD in Noonan syndrome, another RASopathy, is reported to be around 30% [3]. Dysregulation of Ras-MAPK signaling is also likely to be important for non-syndromic ASD, as it has been demonstrated to be a major signaling network hub upon which ASD-linked genes converge [15].

Legius syndrome is a rare RASopathy resulting from mutations in *SPRED1* (Sprouty Related EVH1 Domain Containing 1) [16], a member of the Spred protein family and negative regulator of Ras. Legius syndrome presents as a milder form of NF1, with ~ 50% of patients meeting clinical criteria for NF1 [17]. A review of the clinical phenotypes of Legius syndrome patients published to date is presented in Additional file 1: Table 1. A *SPRED1* mutation database is maintained at https://databases.lovd.nl/shared/genes/SPRED1 and to date, 287 individuals (all ages) have been reported. Similar to NF1 patients, Legius syndrome patients have café-au-lait spots, axillary or inguinal freckling and macrocephaly, but do not have other NF1 manifestations such as neurofibromas, optic pathway gliomas or malignant peripheral nerve sheath tumors. As with NF1, cognitive phenotypes, including learning disabilities and/or intellectual disabilities, have been reported in most studies of Legius syndrome [16–26]. Developmental delay, ADHD and hyperactivity are also frequently seen [16–18, 20, 21, 27, 28] and autistic features or autism spectrum disorder have been reported in a subset of patients [20, 27, 28]. However due to the small number of patients, it has not been possible to investigate the prevalence and specific characteristics of ASD in Legius syndrome with standard rating scales, as has been done for NF1 and other RASopathies such as Noonan syndrome [3, 5, 6, 13, 14, 29]. A study investigating intelligence and behavior with standard questionnaires in 15 Legius syndrome patients was able to demonstrate that performance IQ is lower in these patients, but was not sufficiently powered to detect if there were significant differences in behavioral sub-scores compared to a NF1 cohort [28].

**Table 1:** Cohorts, number of animals and housing conditions for all behavior experiments

Background strain	Sex	N	Behavior test	Housing	Figure
*Neonatal mice*					
B6	Male Female	WT *n* = 17; *Spred1-/- n* = 12 WT *n* = 7; *Spred1-/- n* = 10	USV at P4-12 Righting reflex P4, P8 (first subgroup only, 12–17/genotype)	With birth litter and dam	Figure 2, Additional file 1: Fig. 3A, B
B6	*Spred1*+/− Female dams for the above neonatal cohort	Naïve *n* = 7; experienced *n* = 5	Note: for experienced dams—2nd litter *n* = 3; 3rd litter *n* = 1; 4th litter *n* = 1		
*Adult mice*					
B6;129T2 F2	Female Male	WT *n* = 8; *Spred1-/- n* = 8 WT *n* = 8; *Spred1-/- n* = 8	Tube test at 9–13 weeks	Couples: WT with *Spred1-/-*	Figure 1A, B
B6;129T2 F2	Male Female	WT *n* = 6; *Spred1-/- n* = 7 WT *n* = 10; *Spred1-/- n* = 7	Open field at 6–15 weeks	Mixed genotype group housing	Additional file 1: Fig. 3C, D
B6	Male	WT *n* = 10; *Spred1+/−* *n* = 10	Tube test at 10–15 weeks	Couples: WT with *Spred1+/−*	Additional file 1: Fig. 1D–F
B6	Female	WT *n* = 10; *Spred1-/-* *n* = 10	Tube test at 11–20 weeks nesting at 14–23 weeks	Couples: WT with *Spred1-/-*	Additional file 1: Fig. 1A–C Figure 1G
B6	Female	WT *n* = 7; *Spred1-/-* *n* = 9	Nesting at 20–35 weeks	Couples: WT with *Spred1-/-*	Figure 1G
B6	Female	WT *n* = 11; *Spred1-/-* *n* = 11	Marble burying at 8–20 weeks	Mixed genotype group housing	Additional file 1: Fig. 2F
B6	Male	WT *n* = 15; *Spred1-/- n* = 14	Marble burying at 9–10 weeks (n = 8/genotype only) USV at 10–11 weeks	Couples: WT with *Spred1-/-*	Additional file 1: Fig. 2F Figure 1C–F
B6	Male	WT *n* = 9; *Spred1-/- n* = 9	Resident intruder at 10–13 weeks	Mixed genotype group housing	Additional file 1: Fig. 1G–H
B6	Female	WT *n* = 15; *Spred1-/- n* = 15	Three-chamber test at 20 weeks (n = 13–14/genotype) burrowing at 28 weeks (n = 8/genotype only) MEK inhibitor + nesting at 40 weeks. (WT + DMSO *n* = 8; WT + PD325901 *n* = 7; *Spred1-/-* + DMSO *n* = 7; *Spred1-/-* + PD325901 *n* = 8)	Mixed genotype group housing	Additional file 1: Fig. 2A–E Additional file 1: Fig. 2G Figure 4
*MEK inhibitor* + *Automated tube test*					
B6: WT + DMSO vs *Spred1-/-* + DMSO	Male	WT + DMSO *n* = 4; *Spred1-/-* + DMSO *n* = 4	Tube test at 11–18 weeks	Couples: WT + DMSO with *Spred1-/-* + DMSO	Additional file 1: Fig. 4A–C
B6: WT + DMSO vs *Spred1-/-* + DMSO	Female	WT + DMSO *n* = 4; *Spred1-/-* + DMSO *n* = 4	Tube test at 10–11 weeks	Couples: WT with *Spred1-/-*	Additional file 1: Fig. 4D–F
B6: WT + DMSO vs *Spred1-/-* + PD325901	Female	WT + DMSO *n* = 8; *Spred1-/-* + PD325901 *n* = 8	Tube test at 10–12 weeks (cohort1), 23 weeks (cohort2)	Couples: WT + DMSO with *Spred1-/-* + PD325901	Figure 3A; Additional file 1: Fig. 5A
B6: WT + DMSO vs WT + PD325901	Female	WT + DMSO *n* = 8; WT + PD325901 *n* = 8	Tube test at 11–15 weeks	Couples: WT + DMSO with WT + PD325901	Figure 3C; Additional file 1: Fig. 5C
B6: *Spred1-/-* + DMSO vs *Spred1-/-* + PD325901	Male	*Spred1-/-* + DMSO *n* = 6; *Spred1-/-* + PD325901 *n* = 6	Tube test at 10–18 weeks	Couples: Spred1-/- + DMSO with *Spred1-/-* + PD325901	Figure 3B; Additional file 1: Fig. 5B

In addition to the clinical overlap between NF1 and Legius syndrome, at the biochemical level the SPRED1 protein interacts with neurofibromin, recruiting neurofibromin to the membrane and enabling Ras-GAP activity of neurofibromin and suppression of downstream ERK activation [23, 30]. Pathogenic missense mutations in the EVH1 (neurofibromin-binding) domain of SPRED1 result in decreased affinity for neurofibromin and attenuation of ERK suppression [23]. The biochemical and clinical overlap between NF1 and Legius syndrome suggests that *Spred1* mouse models can offer insights into the shared molecular mechanisms underlying both of these syndromes. Further bolstering this hypothesis, a recent clinical study compared the ASD phenotype across three RASopathies, NF1, Noonan syndrome and cardiofaciocutaneous syndrome (CFC), which each stem from mutations in different regulators of the RAS-MAPK pathway. This revealed that ASD profile was relatively similar across RASopathies [4]. There were significant differences in developmental delay and trajectory between RASopathies but Autism Diagnostic Observation Schedule (ADOS) scores were not significantly different, suggesting that some generalizability of mechanistic aetiology over RASopathies may be possible.

Mouse models for RASopathies consistently exhibit cognitive deficits, modelling some aspects of patient cognitive phenotypes [31]. *Nf1+/−*  and *Spred1-/-* mice have impaired hippocampal-dependent spatial memory in the Morris water maze [32–34] and *Nf1+/−* mice have working memory dysfunction [35]. *Spred1-/-* mice also have decreased performance in visual discrimination learning [32] and impaired instrumental operant learning [36]. Consistent with these alterations in hippocampus-dependent behaviors, defects in hippocampal LTP are seen in both *Nf1+/−*  and *Spred1-/-* mice [32, 34]. *Nf1+/−* mice have impairments in long term social recognition memory, but their short term preference for social novelty is intact [37, 38]. However, it is not known if *Spred1-/-* mice can also model social and behavior alterations relevant to ASD.

Here, we examined ASD-linked behaviors in a *Spred1-/-* mouse model for Legius syndrome. *Spred1-/-* mice exhibited abnormal social behavior in the automated tube test and impaired nest building behavior. Ultrasonic vocalization communication was altered in *Spred1-/-*, both in early neonatal development and in adulthood in response to social stimuli. Acute pharmacological inhibition of the Ras-MAPK pathway with a MEK inhibitor in adulthood reversed abnormal social dominance behavior and nesting impairments in *Spred1-/-*, pointing to a key role for dysregulated Ras-MAPK signaling in regulating social behavior.

## Methods

### Animals

*Spred1-/-* mice were generated as previously described [39]. *Spred1+/−*  mice were backcrossed at least 10 times onto the C57BL/6 J (B6) background prior to breeding to generate experimental animals. In two separate cohorts of mice for the automated tube test and open field (see Table 1), *Spred1+/−*  mice on B6 background were crossed with 129T2/SvEmJ mice, and F2 hybrids (referred to as B6;129T2 from hereon) from these crosses were used for experiments. Mice were genotyped by PCR of DNA extracted from neonatal toe biopsy. For all behavior experiments analyzing *Spred1-/-* mice, *Spred1* + */* + wildtype mice (henceforth referred to as WT), which were littermates from the *Spred1+/−* x *Spred1+/−*  breeding, were used as controls. Mice were housed under standard laboratory conditions on a 12 h dark/light cycle and all behavior testing was carried out in the light cycle. Bedding was Lignocel® BK 8/15, and standard laboratory food and water were available ad libitum. The details on the cohorts of mice for all behavior tests are summarized in Table 1, with information on background strain, sex, age, housing and any drug treatment. All experiments were reviewed and approved by the Animal Ethics Committee of KU Leuven or the Animal Ethical Committee of Erasmus MC Rotterdam in accordance with the European Union Directive 2010/63/EU.

### Automated tube test

Mice were weaned at 3–4 weeks and housed in same sex pairs, with one WT and one *Spred1-/-* mouse together (Table 1). Occasionally when weaning, a correct genotype/sex partner was not immediately available. In that case, the mouse was group housed with same sex littermates until the next litter was weaned, and then paired appropriately. If this housing rearrangement was done, it was done no later than 6 weeks of age. Testing was carried out in adult mice in age matched cohorts, starting at 2–3 months of age. Mice were behaviorally naïve before tube test experiments and were not used for other behavior experiments, so as not to interfere with social hierarchy. The automated tube test apparatus (Benedictus Systems, Rotterdam, The Netherlands) has been previously described [40, 41]. In brief, it is a transparent 50 cm long fiberglass tube with a center door, connected at both ends to fiberglass boxes with automated entrance doors, and an infrared tracking system. Training and tournament protocols were carried out based on previous studies [40]. A 5-day training protocol was first performed for all mice. On day 1 mice received 2 habituation trials with doors open, allowing free exploration of the tube for a maximum of 180 s, or until the mice walked through the tube into the other box. On days 2–5, mice had a limit of 30 s to walk through the tube, after which they were assisted manually. If the mice remained in the starting box for more than 5 s, they received an air puff to assist them entering the tube. They received 6 trials total each day, starting from alternate boxes. Two days after training was complete, the tournament protocol was started and lasted for 5 days. On each tournament day, mice received 2 training trials, 45 min before the tournament matches started. In the matches, a mouse was placed in each box, and the doors automatically opened simultaneously, allowing mice to move into the tube. If the mouse remained in the box for more than 5 s, it received a puff of air. The center door opened automatically when both mice were within 4 cm of the door. The match was complete when one mouse had retreated with all 4 paws into its starting box (assigned the “loser”), and the other mice was assigned as the “winner”. The apparatus was cleaned after every training trial and tournament match with a 70% ethanol solution. For all tournaments, cohorts of 4–6 cages of same sex pairs (*n* = 4–6 mice/genotype) played against each other. Both male and female cohorts were tested on B6 and B6;129T2 F2 hybrid backgrounds. For cohorts on the B6;129T2 background, matches were played between all mice and WT-*Spred1-/-* matches are analyzed and presented. For all other cohorts including MEK inhibitor studies, matches were played only between WT and *Spred1-/-* mice in each cohort. For MEK inhibitor studies, cohorts of both male and female mice were used. On each tournament day, the same matches were played, but with a pseudorandomized order, with starting box alternated and inter-match interval balanced. Experiments were repeated on independent cohorts to verify reproducibility.

### Neonatal ultrasonic vocalization induced by maternal separation

For pups, the day of birth was defined as postnatal day 0 (P0). Pups were evaluated for ultrasonic calls emitted at neonatal stages on P4, P6, P8, P10 and P12. These mice were a separate cohort not used for adult behavior assays. On each day of testing, pups were individually removed from the nest and placed in an empty plastic container located inside a sound attenuating Styrofoam box. An ultrasound microphone, sensitive to frequencies of 10–180 kHz (Avisoft UltrasoundGate condenser ultrasound microphone CM16, Avisoft Bioacoustics e.K., Glienicke/Nordbahn, Germany), was placed through a hole in the Styrofoam box approximately 20 cm above the pup. Ultrasonic vocalizations (USVs) were recorded for 3 min using Avisoft-RECORDER software, using a 250 kHz sampling rate and 16-bit format. After testing, pups were weighed and returned to the nest. After testing on P4, pups were also individually identified with a paw tattoo using a 22G syringe needle filled with Indian ink. Two independent cohorts were analyzed. In the second cohort, righting reflex was also assessed in mice on P4 and P8, after USV recording. Pups were placed on their back and the time taken to right onto all 4 paws was recorded. Data from both sexes was pooled as we saw no significant differences in vocalization between sexes (data not shown). We also excluded dam experience as a major factor influencing our data (data not shown), and present here data pooled from pups born to both naïve and experienced dams.

### Adult ultrasonic vocalization to social stimuli

USVs during courtship behavior were measured in a separate cohort of adult male mice, aged 10-11 weeks, in response to unfamiliar female adult intruders. Male adult subject mice were housed overnight with an adult female B6 for sexual experience. They were then housed individually for 3 days. Adult female B6 mice (aged 2–3 months) that served as stimuli were different to the mice used for overnight housing, and were group housed [4–5 mice per cage]. Female mice did not have estrus induced, and estrous cycle was not tracked. On the day of the test, the male subject was placed in a clean testing cage, in a sound attenuating Styrofoam box with an ultrasound microphone above, as described above. The mice were habituated for 10 min to the novel environment. In the last 4 min, a baseline recording was made. Next, an unfamiliar female mouse was introduced into the cage and the mice were allowed to freely interact whilst USVs were recorded for 4 min. We did not determine whether the recorded USVs were from male or female mice, because in the context of a male–female encounter, likely representing courtship USVs, USVs are mainly produced by males [42, 43].

### USV analysis

Acoustic properties of audio files were analyzed in Avisoft SASLab Pro software (version 5.2.10, Avisoft Bioacoustics e.K., Glienicke/Nordbahn, Germany). Spectrograms were generated with a Fourier transformation (FFT)-length of 1024 and time window overlap of 75% (100% frame, Hamming window). A high-pass filter at 30 kHz cut-off was applied to reduce background noise outside of the relevant frequency band. Call detection was by an automatic threshold-based algorithm and a hold-time mechanism (hold time 20 ms). An experienced user blinded to genotype checked accuracy of the call detection to ensure concordance between automated and observational detection. Parameters automatically extracted for each file included number of calls, the mean duration of calls, mean peak frequency (pitch, kHz) and mean peak amplitude (loudness, measured in decibels) [44]. For analysis of neonatal stages, no sex differences were observed (data not shown), so data from male and female pups were pooled together for analysis.

### Three chamber test for sociability and preference for social novelty

Female WT and *Spred1-/-* mice were tested at 20 weeks of age (Table 1). The apparatus was an enclosed rectangular transparent Plexiglas box with opaque floor (width x depth x height: 94 × 26 × 30 cm), divided into three chambers. The central chamber (42 × 26 cm) was connected to a left and right chamber (26 × 26 cm) via openings (6 × 8 cm) in that could be manually closed. The left and right chamber contained cylindrical wire cups (height x diameter: 11 × 10 cm). Access to the chambers was controlled by manually operated guillotine doors. The test consisted of three phases, conducted consecutively in a three-chambered test apparatus as previously described [45]. In the habituation phase [5 min], mice were placed in the central chamber to acclimatize. In the sociability phase (10 min), the animal’s interest in a social stimulus (a same sex conspecific, not used for behavior testing) versus an empty chamber were tested, with the social stimuli mouse constrained under a wire cage in one chamber, and an empty wire cage in the other chamber. In the final stage, preference for social novelty (10 min), interest in a familiar social stimulus versus a non-familiar social stimulus was assessed. The apparatus was cleaned in between subjects with 70% ethanol. Mice were recorded from above with a camera and their heads tracked using ANY-Maze software (Stoelting Europe, Ireland). Sniffing time (= time spent within 2 cm proximity to the wire cage) and pathlength were quantified. For the sociability phase, a social preference index was calculated as follows: Time_stranger1_/(Time_stranger1_ + Time_empty_).

### Resident intruder

Intruder mice (B6 adult males) were individually housed for 9 days, and were age matched to residents. Intruders were used only once. Each male resident mouse (*Spred1-/-* and WT) was housed for 1 week prior to the test with one adult B6 female mouse, without changing the bedding. On the day of the test, the intruder mouse was introduced into the cage of the resident and female and behavior was scored for 30 min before the intruder was removed. The latency until an attack occurred, the latency between attacks and the total number of attacks were measured.

### Open field

Open field was performed in male and female mice on the B6;129T2 background. The open field was conducted in a circular arena with a diameter of 110 cm. Mice were placed gently in the center of the arena and allowed to freely explore for 10 min. The arena was cleaned with 70% ethanol in between subjects. Mice were recorded from above with a camera and their body position tracked with the SMART video tracking system (Panlab, Barcelona, Spain). For analysis, the arena was divided into three regions: inner region of 25 cm diameter, surrounded by a middle region 10 cm radius and then an outer region of 10 cm radius. Total path length and percentage of time spent in each region were calculated. No sex differences were observed, therefore data from both sexes was pooled for analysis.

### Nesting behavior

Nest building was conducted based on a previously reported protocol [46]. One week before testing, mice were single housed in standard Makrolon 3108 plastic mouse cages, with 5 cm deep clean bedding material but without environmental enrichment items to habituate. The testing phase was a 2-day protocol. On day 1 mice received pressed cotton nestlets weighing a total of 3 g at noon. Nest building behavior was assessed on day 2, in the morning. For qualitative assessment, a 5-point rating scale was used as previously published [46], with a score of “1” representing largely untouched nesting materials (no shredding) and “5” indicating a perfect nest with high sides.

### Marble burying

Mice were habituated for 10 min in a large Makrolon 3108 plastic mouse cage (375 × 215 × 140 mm) with 5 cm depth clean bedding material (Lignocel® BK 8/15). Subsequently, marble burying was tested for 40 min. During the test phase, cages contained 5 cm deep clean bedding material with 10 glass marbles (14 mm diameter) arranged in a 2 × 5 grid on top of the bedding. 10 marbles were used instead of 20 to reduce the impact of overexposure to simultaneous stimuli. The number of buried marbles was counted. A marble was consider buried when 2/3 of the marble was covered by bedding.

### Burrowing

Burrowing is an innate behavior, described in this experiment as ‘digging a hole or tunnel in or through food pellets in a tube’. The experiment was carried out based on a previously reported protocol [47]. In the habituation phase on day 1, a full burrow tube is placed overnight into the group home cage of the mice, which are later individually tested. Each burrow is filled with 200 g food pellets normally supplied as diet. The testing phase started on day 2 around 5 pm, as mice are normally awake at this time and burrowing starts more slowly than during the dark phase when mice are very active. Mice were placed individually in Makrolon 3108 plastic mouse cages together with a filled burrow tube. Two hours after the start, a snapshot measurement was taken of the weight of the remaining food pellets; the burrow was emptied into a container and weights, then the pellets were replaced into the burrow and placed back into the cage. If the mouse was in the burrow, it was gently removed and placed in the cage. The weight of non-burrowed pellets was presented as a percentage of total weigh of 200 g food pellets. The second reading was taken the next morning at 9am, again weighing the remaining non-burrowed food pellets.

### MEK inhibitor treatment

The MEK inhibitor PD325901 (Selleckchem, Munich, Germany) was dissolved in dimethyl sulfoxide (DMSO) to a final concentration of 5 mg/ml and frozen into aliquots, which were defrosted as needed immediately prior to injection. For all studies, animals in the treated group received 5 mg/kg PD325901, whilst the vehicle group received DMSO alone. This dose was chosen based on its effectiveness in attenuating ERK phosphorylation in mice [48, 49] and at reversing other phenotypes in RASopathy mouse models [50, 51]. Injection volumes were 1 µl per g (mouse weight). Mice were injected intraperitoneally on alternating sides each day with 0.3 ml insulin syringes (BD Micro-Fine). For automated tube test experiments, drugs were injected daily starting 3 days prior to the first day of training and continuing throughout training and tournaments, and injections were 5–6 h before behavior testing started each day. This regimen was based on previous studies that successfully reversed behavioral phenotypes in RASopathy mice [52, 53]. For each different tube test experiment (WT + DMSO vs *Spred1-/-* + DMSO; *Spred1-/-* + DMSO vs *Spred1-/-* + PD325901; WT + DMSO vs *Spred1-/-* + PD325901 and WT + DMSO vs WT + PD325901), a new, separate cohort of mice was used that were naïve to the automated tube test. Mice were always housed in pairs, with one mouse from each ‘treatment’ per cage, as outlined in Table 1. For nest building experiments, mice were injected for 3 days and the nesting protocol initiated 5–6 h after last injection, as described above. After the initial nesting test, mice were left untreated for 3 weeks for drug washout, and then re-tested for nest building. Tube test training/testing each day was started 5–6 h after injection.

### Western blotting

For cohorts of mice used for the MEK inhibition study in the automated tube test, hippocampal lysates were collected for western blot analysis. 1 h after the last automated tube test tournament on day 5, mice were euthanized by cervical dislocation, the brain removed and hippocampi rapidly dissected out and snap frozen in liquid nitrogen. Tissue was homogenized in RIPA buffer (150 mM NaCl, 50 mM Tris–HCl, 1 mM EDTA, 1 mM EGTA, 0.1% Triton X-100, 0.1% Na-deoxycholate) with protease inhibitors (Complete, Roche) and phosphatase inhibitors (PhosphoSTOP, Roche) added. Protein concentration was adjusted to 1 µg/µL and 10 µg protein was loaded onto a NuPAGE Bis–Tris 4–12% mini gel (Invitrogen). The blot was probed with primary antibodies for pERK (Phospho-p44/42 MAPK #4370, anti-rabbit, Cell Signaling Technology) and ERK (p44/42 MAPK #9107, anti-mouse, Cell Signaling Technology). Secondary antibodies were Dylight IR 680 and IR 800 (Thermo Scientific) and were visualized using an Amersham Typhoon Biomolecular Imager. The blot was stripped with Restore™ PLUS Western Blot Stripping Buffer (Thermo Scientific) and re-probed with beta-actin (A5316, anti-mouse, Sigma-Aldrich) as loading control. Quantitative image analysis was performed in ImageJ (v1.51n, NIH) using the Gels function. The ratio of pERK to total ERK was calculated, normalized to a sample from the WT-Vehicle group from DMSO-only treated group (Additional file 1: Supplementary Fig. 4A), and expressed as arbitrary units (a.u.).

### Statistics

For automated tube test experiments, including MEK inhibitor experiments, each day of the tube test was analyzed with two-tailed binomial distribution, with the null hypothesis that if both groups were similar, 50% of matches are won by each group (i.e. chance level of winning). For most other data, including male USV, marble burying, burrowing and western blotting, normality of data was tested with the D’Agostino-Pearson test. If data were normally distributed, a parametric unpaired independent samples *t*-test was used. If data were not normally distributed, non-parametric Mann–Whitney test was performed. Neonatal USVs were analyzed with 2-way ANOVA with repeated measures, with age and genotype as the main factors. Nest building: for naïve mice, an unpaired independent *t*-test was used. For MEK inhibitor experiments assessing nesting, 2-way ANOVA with treatment and genotype as factors was performed. For sociability and social preference, sniff time was analyzed with 2-way ANOVA, using chamber and genotype as factors. Preference index for sociability was analyzed with an unpaired independent *t*-test. A significance level of 0.05 was used for all statistical analyses. Statistical analysis was performed in GraphPad Prism 8 (GraphPad Software, Inc., La Jolla, CA, USA).

## Results

### *Spred1-/-* mice exhibit altered social and communicative behavior

**Fig. 1:**
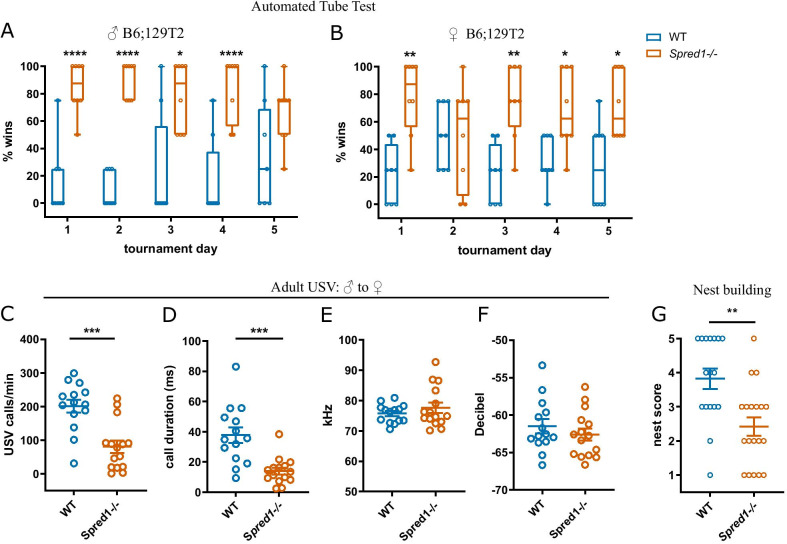
Social and communicative impairments in *Spred1-/-* mice. **A**, **B** Automated tube test for social dominance. **A** Mean percentage of matches won per day in the automated tube test across 5 days of tournaments between WT and *Spred1-/-* mice, 129T2/SEMvJ background. Significant effect of genotype on days 1–4 (Two tailed binomial test compared to chance (50%): day 1 *p* < 0.0001; day 2 *p* < 0.0001; day 3 *p* = 0.011; day 4 *p* < 0. 0001). Box and whisker plots of median and quartiles of 2 independent experiments, pooled; *n* = 8 male mice/genotype. **B** Mean percentage of matches won per day in the tube test across 5 days of tournaments between WT and *Spred1-/-* female mice on the 129T2/SVEmJ background. Significant effect of genotype on days 1, 3, 4 and 5 (Two tailed binomial test compared to chance (50%): day 1 *p* = 0.0011; day 3 *p* = 0.0011; day 4 *p* = 0.0251; day 5 *p* = 0. 01). Box and whisker plots of median and quartiles of 2 independent experiments, pooled; *n* = 8 mice/genotype. **C–F** USVs from WT and *Spred1-/-* male mice (B6 background) to a novel B6 female: **C** Number of USV calls/min made by male mice (unpaired *t*-test, *t* = 4.481, *df* = 27 *p* = 0.0001). **D** Mean call duration of male mice in response to a novel female (Mann Whitney test, *p* = 0.0002). **E** Mean peak frequency of USV calls (unpaired *t*-test, *t* = 1.001, *df* = 27, *p* = 0.3258). **F** Mean peak amplitude of USV calls (unpaired *t*-test, *t* = 0.8998, *df* = 27, *p* = 0.3762). *n* = 14–15 mice/genotype, mean ± SEM. **G** Nest building score of female *Spred1-/-* and WT mice (B6 background) after 24 h, indicating quality of nest building. 1 = no nest shredding; 5 = high-sided nest with all material shredded (unpaired *t*-test, *t* = 3.496, *df* = 34, *p* = 0.0013). *n* = 17–19 mice/genotype, mean ± SEM. For all figures, asterisks indicate: *p < 0.05, **p < 0.01, ***p < 0.001, ****p < 0.0001

To examine whether social behaviors were altered in *Spred1-/-* mice, we evaluated mice in the automated tube test, which measures social dominance [40]. *Spred1-/-* mice on a B6;129T2 background won a majority of matches against wildtype (WT) opponents, a phenotype stable across 5 days of tournaments (Fig. 1A, B). This social dominance phenotype was seen in both male (Fig. 1A) and female (Fig. 1B) *Spred1-/-* mice against WT controls (Males—two tailed binomial test compared to chance (50%): day 1 *p* < 0.0001; day 2 *p* < 0.0001; day 3 *p* = 0.011; day 4 *p* < 0. 0001; Females—two tailed binomial test compared to chance (50%): day 1 *p* = 0.0011; day 3 *p* = 0.0011; day 4 *p* = 0.0251; day 5 *p* = 0.01). As background strain can influence behavior, we verified that this phenotype was robust to background strain, observing that the same social dominance phenotype was observed *Spred1-/-* mice when backcrossed more than 10 generations onto the B6 background in both male (Additional file 1: Supplementary Fig. 4A; two tailed binomial test compared to chance (50%): day 2 *p* = 0.0213; day 3 *p* = 0.0213; day 4 *p* = 0.0213; day 5 *p* = 0.0005) and female cohorts (Additional file 1: Supplementary Fig. 1A; two tailed binomial test compared to chance (50%): day 2 *p* = 0.0013; day 3 *p* < 0.0001; day 4 *p* < 0.0001; day 5 *p* < 0.0001). No genotype differences were seen in time to reach the center door in the training phase of the tube test (Additional file 1: Supplementary Fig. 1B, B6 background; 2 Way ANOVA: effect of day *F*(8,64) = 3.296, *p* = 0.0033; no effect of genotype, no interaction), and body weights were stable throughout the experiment (Additional file 1: Supplementary Fig. 1C, B6 background). The social dominance phenotype was not seen *Spred1+/−*  male mice when playing matches against WT controls in the automated tube test (Additional file 1: Supplementary Fig. 1D–F; Two tailed binomial test compared to chance (50%), days 1–5 *p* > 0.1). We examined whether the social dominance phenotype in male *Spred1-/-* mice was linked to aggression by performing the resident intruder test in a separate cohort of mice. There was no difference in either latency to attack or the total number of attacks that *Spred1-/-* male mice made onto intruders compared to WT littermates (Additional file 1: Supplementary Fig. 1G–H; latency—unpaired *t*-test, *t* = 1.794, *df* = 16, *p* = 0.0918; total attacks—unpaired *t*-test, *t* = 1.386, *df* = 16, *p* = 0.1848). In an open field test *Spred1-/-* mice displayed normal thigmotaxis, suggesting they were not less anxious than WT controls (Additional file 1: Supplementary Fig. 3C). Social approach and social preference in the 3-chamber social interaction test were normal in *Spred1-/-* mice (Additional file 1: Supplementary Fig. 2A–C; sociability phase preference index—unpaired *t*-test, *t* = 0.6898, *df* = 25, *p* = 0.4967. Social preference—2-way ANOVA, effect of chamber side: *F*(1,25) = 6.396, *p* = 0.0181; no effect of genotype). There was a significant reduction in the number of transitions between compartments made by *Spred1-/-* mice in both phases of the test (Additional file 1: Supplementary Fig. 2D–E. Sociability phase—unpaired *t*-test, *t* = 2.805, *df* = 25, *p* = 0.0096. Social preference phase—unpaired *t*-test, *t* = 2.061, *df* = 25, *p* = 0.0499), however locomotor activity in the open field was normal (Additional file 1: Supplementary Fig. 3D). This phenotype is similar to the slightly lower locomotor activity observed in *Spred1-/-* mice in operant chambers [36].

**Fig. 2:**
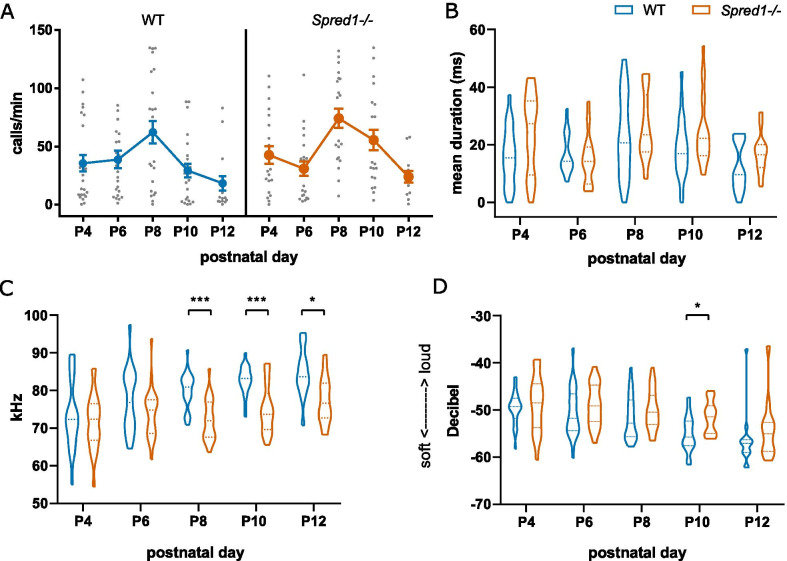
*Spred1-/-* mice exhibit deficits in neonatal ultrasonic vocalization. Neonatal USVs upon maternal separation in WT and *Spred1-/-* mice were recorded from P4 to P12. **A** Mean call rate per minute (2-way ANOVA with repeated measures: main effect of age, *F*(4,156) = 12.28, ****p < 0.0001, no effect of genotype, no interaction). Mean ± SEM. **B** Mean call duration in milliseconds (2-way ANOVA with repeated measures: effect of age, *F*(4,156) = 7.347, *p* < 0.0001; no effect of genotype; no interaction). **C** Mean peak frequency of USV calls (2-way ANOVA with repeated measures: effect of age *F*(4,150) = 5.866, *p* = 0.0002; effect of genotype *F*(1,150) = 55.13, *p* < 0.0001; no interaction). **D** Mean peak amplitude of USV calls (2-way ANOVA with repeated measures: effect of age *F*(4,205) = 10.77, *p* < 0.0001, effect of genotype *F*(1,205) = 9.181, *p* = 0.0028; no interaction). For all analyses, *n* = 22–24 mice/genotype. Violin plots show median and quartiles. For **C** and **D**, asterisks indicate post-hoc Bonferroni multiple comparisons tests: *p < 0.05, **p < 0.01, ***p < 0.001

**Fig. 3:**
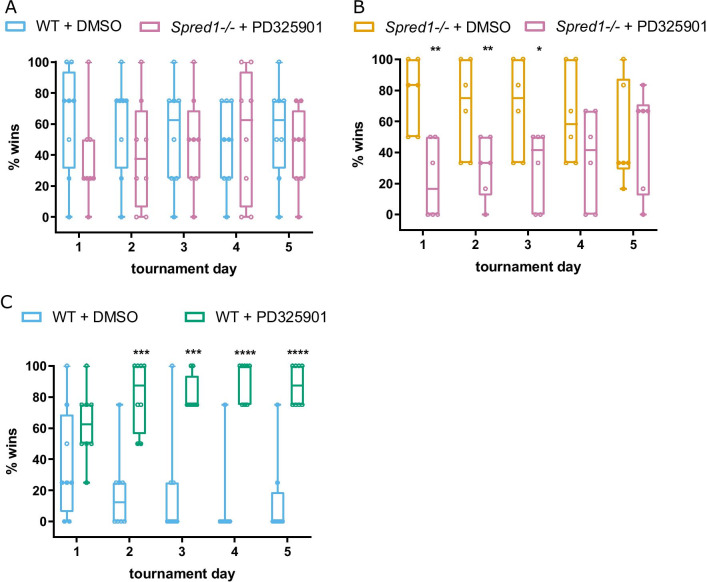
MEK inhibition abolishes the social dominance phenotype in *Spred1-/-* mice. **A** Mean percentage of matches won per day in the tube test across 5 days of tournaments between WT treated with DMSO vehicle and *Spred1-/-* mice treated with PD325901. Two tailed binomial tests compared to chance (50%): no significant differences day 1–5. *n* = 8 mice/genotype. **B** Mean percentage of matches won per day in the tube test across 5 days of tournaments between *Spred1-/-* treated with DMSO vehicle and *Spred1-/-* mice treated with PD325901, with significant differences on day 1–3 (Two tailed binomial test compared to chance (50%): day 1 *p* = 0.0011; day 2 *p* = 0.0011; day 3 *p* = 0.025). *n* = 6 mice/genotype. **C** Mean percentage of matches won per day in the tube test across 5 days of tournaments between WT treated with DMSO vehicle and WT mice treated with PD325901. Two tailed binomial tests compared to chance (50%): significant differences day 2–5; Two tailed binomial tests compared to chance (50%): day 2 *p* = 0.0003; day 3 *p* = 0.0003; day 4 *p* < 0.0001; day 5 *p* < 0.0001. *n* = 8 mice/genotype. All graphs are box and whisker with median and quartiles. *p < 0.05, **p < 0.01, ***p < 0.001, ****p < 0.0001

To study social communication in *Spred1-/-* and WT mice, male ultrasonic vocalization (USV) during an encounter with a novel B6 female conspecific was measured. Male *Spred1-/-* mice (B6 background) emitted significantly fewer USVs in response to a female compared to WT littermate controls (Fig. 1C; unpaired *t*-test, *t* = 4.481, *df* = 27 *p* = 0.0001). They also emitted on average shorter duration calls compared to WT (Fig. 1D; Mann Whitney test, *p* = 0.0002). The average pitch (mean peak frequency) and amplitude of calls (mean peak amplitude) was not significantly altered (Fig. 1E-F; frequency—unpaired *t*-test, *t* = 1.001, *df* = 27, *p* = 0.3258; amplitude—unpaired *t*-test, *t* = 0.8998, *df* = 27, *p* = 0.3762). Innate behaviors such as nest-building contribute to wellbeing and support social functions in mice [54], therefore nest building behavior was examined in adult female *Spred1-/-* and wildtype littermate controls. *Spred1-/-* had reduced nesting behavior, with a significantly reduced nesting score compared to WT mice, an indication of the quality of nest building (Fig. 1G; unpaired *t*-test, *t* = 3.496, *df* = 34, *p* = 0.0013). To investigate repetitive behaviors in this mouse line, behaviors that require digging were assessed, as digging has been interpreted as a repetitive behavior. No genotype differences were seen in the percentage of marbles buried between WT and *Spred1-/-* mice (Additional file 1: Supplementary Fig. 2F. Males: unpaired *t*-test, *t* = 0.4545, *df* = 14, *p* = 0.6564. Females: unpaired *t*-test, *t* = 0.8402, *df* = 20, *p* = 0.4107). Burrowing behavior was assessed as a measure with more ecological validity than marble burying, as burrowing behavior is seen in wild rodents [47, 54]. The amount of food pellets burrowed after either 2 h or overnight, was also unchanged in *Spred1-/-* mice (Additional file 1: Supplementary Fig. 2G. 2-way ANOVA with repeated measures, *F* < 1.00; *p* > 0.1). Thus, *Spred1-/-* have impaired social behavior and communication but no digging phenotypes that would be representative of changes in repetitive behaviors.

### Social communication deficits in *Spred1-/-* mice arise neonatally

We next asked whether USV deficits are already present in early neonatal development in *Spred1-/-* mice. Isolation-induced ultrasonic vocalizations in *Spred1-/-* and WT pups (on the B6 background) were measured from P4-12. There were no significant differences between sexes at any of the time points, therefore data from male and female pups were pooled together for analysis. As previously reported in the literature for B6 mice [55], the rate of vocalization peaked at P8 in WT mice, and then declined to low levels again by P12 (Fig. 2A. 2-way ANOVA with repeated measures: main effect of age, *F*(4,156) = 12.28, *p* < 0.0001, no effect of genotype). No main effect of genotype was seen on call rate (Fig. 2A) or call duration (Fig. 2B; 2-way ANOVA with repeated measures: effect of age, *F*(4,156) = 7.347, *p* < 0.0001; no effect of genotype). Analysis of spectral properties of *Spred1-/-* and WT USVs revealed a main effect of genotype on call pitch (Fig. 2C; 2-way ANOVA with repeated measures: effect of age *F*(4,150) = 5.866, *p* = 0.0002; effect of genotype *F*(1,150) = 55.13, *p* < 0.0001), with post-hoc comparisons revealing significant decreases in mean peak frequency in calls from *Spred1-/-* pups compared to WT from P8-P12. There was also a main effect of genotype on the loudness in decibels of the call in *Spred1-/-* compared to WT, with post-hoc analysis revealing significant increase in amplitude at P10 (Fig. 2D; 2-way ANOVA with repeated measures: effect of age *F*(4,205) = 10.77, *p* < 0.0001, effect of genotype *F*(1,205) = 9.181, *p* = 0.0028). No main effect of genotype was seen on body weight (Additional file 1: Supplementary Fig. 3A; 2-way ANOVA with repeated measures: main effect of age, *F*(4,159) = 1325, *p* < 0.0001, no effect of genotype). No genotype differences were observed in righting reflex at P4 or P8 (Additional file 1: Supplementary Fig. 3B; 2-way ANOVA with repeated measures: main effect of age, *F*(1,29) = 77.15, *p* < 0.0001; no effect of genotype), indicating intact motor abilities in *Spred1-/-* pups. Together these data indicate altered spectral vocalization properties in neonatal *Spred1-/-* mice that primarily emerge in the second neonatal week.

### Acute pharmacological inhibition of the Ras-MAPK pathway can ameliorate social phenotypes in *Spred1-/-* mice

**Fig. 4:**
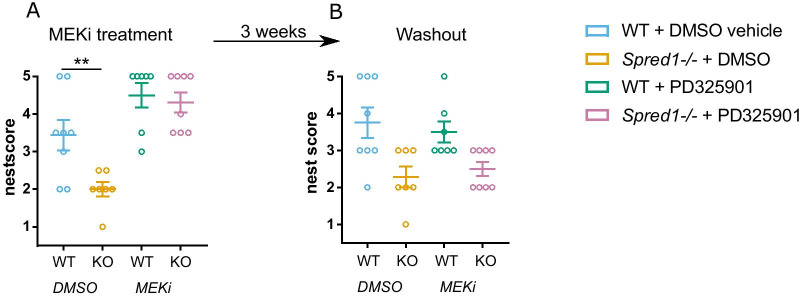
MEK inhibition rescues impairments in nesting behavior in *Spred1-/-* mice. **A** WT and *Spred1-/-* mice were treated with PD325901 or DMSO vehicle for 3 days, then nest building was assessed using a qualitative nest building score. PD325901 significantly increased nesting score in *Spred1-/-* mice (2-way ANOVA: effect of PD325901 treatment *F*(1,26) = 28.81, *p* < 0.0001; effect of genotype *F*(1,26) = 6.679, *p* = 0.0157; no interaction). Post-hoc comparisons show a significant difference between WT + DMSO and *Spred1-/-* + DMSO groups (Sidak multiple comparison test, **p = 0.0066), whereas no difference was seen between WT + PD325901 and *Spred1-/-* + PD325901 groups (Sidak multiple comparison test, *p* = 0.8955). **B** After a 3-week washout period, nest building was assessed again (2-way ANOVA: no effect of treatment, effect of genotype *F*(1,26) = 15.99, *p* = 0.0005; no interaction). Mean ± SEM, *n* = 7–8 mice/genotype

Overactivation of Ras-MAPK signaling is a hallmark of RASopathy disorders. We next asked whether inhibition of downstream MAPK signaling could restore social behaviors in *Spred1-/-* mice. PD325901 is a potent MEK inhibitor that has been demonstrated to shrink neurofibromas both in *Nf1* mouse models [50] and early stage clinical trials in human NF1 patients [56]. PD325901 crosses the blood–brain barrier, as evidenced by reports of human neurotoxicity [57], and further confirmation has been seen in mice, where peripheral administration of PD325901 has been demonstrated to abolish cocaine-induced ERK activation in the striatum [48] and to reduce hyperactive ERK phosphorylation in the hippocampus in a *HRas*^*G12V*^ RASopathy mouse model [49]. To test the effect of PD325901 on social behavior in adulthood, tube test experiments were performed in *Spred1-/-* and WT mice on the B6 background acutely treated with either PD325901 or DMSO vehicle control. All cohorts learned to run through the tube in training sessions (Additional file 1: Supplementary Fig. 4B, Additional file 1: Supplementary Fig. 4E, Additional file 1: Supplementary Fig. 5 A–C). When *Spred1-/-* mice treated with PD325901 played matches against WT mice receiving vehicle, the dominant winning phenotype of *Spred1-/-* mice in the tube test was abolished, with winning at chance level (Fig. 3A). Confirming that PD325901 normalizes social dominance behavior of *Spred1-/-* mice under different social conditions, we saw that *Spred1-/-* mice treated with PD325901 lost significantly more matches against *Spred1-/-* receiving vehicle, an effect that was stable across the first 3 days of tournaments (Fig. 3B; two tailed binomial test compared to chance (50%): day 1 *p* = 0.0011; day 2 *p* = 0.0011; day 3 *p* = 0.025). Tube test tournaments when both *Spred1-/-* and WT mice received vehicle control recapitulated the phenotype of naïve *Spred1-/-* mice over WT mice (Additional file 1: Supplementary Fig. 4A, 4D. Males—two tailed binomial test compared to chance (50%): day 2 *p* = 0.0213; day 3 *p* = 0.0213; day 4 *p* = 0.0213; day 5 *p* = 0.0005. Females—two tailed binomial test compared to chance (50%): day 2 *p* < 0.0001; Day 4 *p* = 0.005; Day 5 *p* = 0.0042), demonstrating that treatment with vehicle alone did not significantly influence behavior. Notably, WT mice were treated with PD325901, they won significantly more matches against WT mice treated with vehicle (Fig. 3C. Two tailed binomial tests compared to chance (50%): day 2 *p* = 0.0003; day 3 *p* = 0.0003; day 4 *p* < 0.0001; day 5 *p* < 0.0001). This indicated a drug x genotype interaction, whereby in *Spred1-/-* mice the effect of PD325901 was to reduce winning, but in WT mice PD325901 had the opposite effect, increasing winning. No differences between groups were observed in weight across the experiment (Additional file 1: Supplementary Fig. 4C, F; Additional file 1: Supplementary Fig. 5 A–C), and we confirmed that in all cohort configurations, PD325901 treatment significantly attenuated hippocampal ERK activation compared to vehicle treatment, verifying the specificity of drug effect (Additional file 1: Supplementary Fig. 6A–D. WT + vehicle vs *Spred1-/-* + vehicle cohort—unpaired *t-*test, *t* = 0.1777, *df* = 8, *p* = 0.8634. WT + vehicle vs *Spred1-/-* + PD325901 cohort—unpaired *t*-test, *t* = 19.69, *df* = 6, *p* < 0.0001. *Spred1-/-* + vehicle vs *Spred1-/-* + PD325901 cohort—unpaired *t*-test, *t* = 3.846, *df* = 10, *p* = 0.0032. WT + vehicle vs WT + PD325901—unpaired *t*-test, *t* = 6.957, *df* = 6, *p* = 0.0004). In accordance with previous studies [32, 36], we do not observe significant differences in total hippocampal pERK levels in *Spred1-/-* mice compared to WT. Acute MEK inhibition also significantly improved nesting behavior in *Spred1-/-* mice, increasing nest building scores to levels similar to WT mice (Fig. 4A; 2-way ANOVA: effect of PD325901 treatment *F*(1,26) = 28.81, *p* < 0.0001; effect of genotype *F*(1,26) = 6.679, *p* = 0.0157; no interaction). After a 3-week washout the effect of PD325901 on *Spred1-/-* nesting behavior was largely abolished, further demonstrating the causality of the MEK inhibition on the behavioral rescue (Fig. 4B; 2-way ANOVA: no effect of treatment, effect of genotype *F*(1,26) = 15.99, *p* = 0.0005; no interaction). Together these results show that acute treatment with PD325901 reverses social behavior phenotypes in adult *Spred1-/-* mice.

## Discussion

This study demonstrated that social behavior impairments in a mouse model for RASopathy disorders could be acutely rescued by pharmacological MEK inhibition. A *Spred1-/-* mouse model for Legius syndrome exhibits a phenotype which models social phenotypes linked to ASD, with enhanced social dominance and deficits in USV communication, as well as impaired nesting behavior. Critically, we could rescue the tube test and nest building social phenotypes of *Spred1-/-* in adulthood by acute MEK inhibition, the first report of a drug targeting the Ras-MAPK pathway rescuing social behavior in a mouse model for ASD. These findings indicate a crucial role for Ras-MAPK signaling in regulating social behaviors.

The social dominance phenotype observed in *Spred1-/-* mice was observed in both sexes, and was also robust to background strain. Hence, we posit that social alterations observed in *Spred1-/-* mice are a robust phenotype suitable for preclinical therapeutic drug discovery. Although Legius syndrome patients carry heterozygous loss-of-function mutations in *SPRED1* [16], we did not observe social dominance alterations in *Spred1+/−*  mice. Previous work has shown that *Spred1* + */ − *mice are mildly impaired in visual discrimination learning, with a less severe cognitive phenotype than *Spred1-/-* mice [32], suggesting that relevant cognitive phenotypes are more clearly modeled in a homozygous mouse model. There is a precedent for this, as although many disorders that feature ASD arise from heterozygous mutations, robust phenotypes are frequently only seen in mice homozygous for the mutation, such as has been observed in mouse models for autism-associated gene *SHANK2* [58, 59].

Notably, the increased social dominance phenotype observed in *Spred1-/-* mice is highly similar to increased social dominance observed in the tube test in several other mouse models for monogenic forms of ASD, including several different *Shank3* mouse models [60, 61], *Shank2*^*Δ6−7*^ mice [62] and *Fmr1 y/-* mice [40]. Increased winning in the tube may model an inability to recognize the social status of conspecifics. Although we see abnormal social dominance behavior in the tube test across multiple days of testing, *Spred1-/-* mice do not show any phenotype in the three-chamber test for sociability, neither in the social approach or social preference phases of the test. This is consistent with studies of other mouse models for RASopathies, where normal social approach behaviors have been seen in *Raf1*^*L613V*^ mice, *KRas*^*G12V*^ mice and *Nf1+/−*  mice [37, 63, 64]. Social preference is also normal in *Nf1*+/− mice [37], but long term social memory after 24 h is impaired in *Nf1+/−*  [37, 38]. Critically, no differences were seen in aggressive behavior in the resident intruder test in male *Spred1-/-* mice, suggesting that the social dominance phenotype seen is not a result of hyper-aggression, nor was there a low anxiety phenotype that would explain the increased dominance. The tube test paradigm measures a more complex social scenario compared to the three-chamber test. Mice meet multiple conspecifics separately, developing and maintaining a hierarchical relationship over time. Social dominance has not yet been examined in other RASopathy mouse models, and our results in *Spred1-/-* mice suggest this test might enable detection of social phenotypes in models for which social approach phenotypes are not seen.

Complementary to alterations in social behavior in the tube test, we saw deficits in USV communication in *Spred1-/-* mice. This phenotype emerged already neonatally, with significant alteration of spectral properties of vocalizations. The neonatal phenotype was similar to the changes seen in USV production in *Nf1+/−*  pups, where lower pitch of USV calls are also seen [65]. Additionally, adult vocalization during social courtship situations was significantly reduced in *Spred1-/-* male mice, indicating that communication specific to a social behavior is disrupted. These results point to *Spred1-/-* as a model for social and communication phenotypes in ASD and RASopathies. Together with the previously reported impairments in cognitive function [32, 36], our data indicate that this mouse model can recapitulate key neurological aspects of the Legius syndrome phenotype.

In addition to altered social and communicative behaviors, ASD is also characterized by restricted or repetitive patterns of behavior, or restricted interests [2]. We did not observe any changes in digging behaviors in *Spred1-/-* mice. Though tests for digging behaviors are commonly used in preclinical ASD research for assessing repetitive behaviors [66], their relevance to restrictive and repetitive behaviors is not clear. Characterization of the ASD phenotype in NF1-ASD patients has shown that the most severe impairments are in the domain of social cognition [14]. Although resistance to change was seen in patients in that study, ASD-typical stereotypical behaviors were not observed [14]. Larger studies of NF1-ASD will be necessary to better understand the differences in NF1-ASD compared to non-syndromic ASD. However, the lack of an overt repetitive behavior phenotype in *Spred1-/-* mice would seem to fit with a NF1-ASD phenotype that does not feature strong impairments in restricted and repetitive behaviors.

To our knowledge this is first demonstration that a specific MEK inhibitor could rescue abnormal social behavior in a mouse model for ASD. Neonatal treatment with PD325901 in other RASopathy mouse models have shown dramatic effects, rescuing growth defects and craniofacial features in a *Raf*^*L613V*^ mouse model [51], and reversing defects in brain morphology and motor learning in a *Nf1*^*GFAP*^cKO mouse model [67, 68]. We show here that with acute treatment in adulthood, enhanced social dominance and nest building deficits can be normalized in *Spred1-/-*. However, in a separate study, impairments in operant learning exhibited by *Spred1-/-* mice could not be reversed by acute adult PD325901 treatment [36]. Similarly, in two other mouse models for RASopathies, *HRas*^*G12V*^ and *KRas*^*G12V*^ mice, learning and memory defects could also not be rescued by PD325901 inhibition in adulthood [49, 64]. This suggests that social behaviors in RASopathies may be more amenable to modulation with MEK inhibition compared to cognitive behaviors, which has implications for clinical translation.

Our data also point to a key role for Ras-MAPK signaling in regulation of social behavior. Many ASD risk genes converge on Ras-MAPK signaling [15], and dysregulated ERK signaling is seen in other forms of syndromic ASD [69] as well as idiopathic ASD [70], suggesting Ras regulation of social behavior may also extrapolate to models for other forms of monogenic ASD. It was previously shown that overexpression of Ras in excitatory neurons can alter social dominance behavior [71]. Here we observed that in the tube test for social dominance, MEK inhibition in WT mice increased social dominance behavior. This indicates that the effect of MEK inhibition was not additive, but modified by interaction with *Spred1* genotype. This points to a scenario whereby modulation of Ras-MAPK signaling away from a defined equilibrium in either direction impacts social functioning. This phenomenon is likely due to specific roles of Ras-MAPK signaling in different neuronal cell types that are important for mediating social behaviors. Cognitive deficits in *Nf1+/−*  mouse models have been demonstrated to be specifically due to loss of *Nf1* in GABAergic neurons [72], with enhancement of inhibitory neurotransmission in *Nf1* mouse models [34] posited to be a main mechanism by which behavior impairments arise. Studies in *Drosophila* have shown that impairments in habituation learning occur both after manipulations that increase Ras-MAPK activity in GABAergic inhibitory neurons, such as loss of function of Spred1 or Nf1, and after manipulations that decrease Ras-MAPK in excitatory neurons [73]. Hence, it will be important in future studies to determine if there is a role for Spred1 in GABAergic neurotransmission and how it might regulate social behavior phenotypes.

In addition to regulation of ERK signaling, Spred1 controls actin polymerization by regulating RhoA-ROCK signaling via a direction interaction with RhoA [74, 75]. Hematopoietic stem cells from *Spred1-/-* mice have enhanced actin polymerization that is dependent on RhoA-ROCK, but not on ERK, RAF or mTOR signaling cascades [74]. Although this mechanism has not yet been demonstrated in neurons, it raises the intriguing possibility that Spred1 regulation of the actin cytoskeleton could be involved in synaptic plasticity mechanisms underpinning behavioral phenotypes observed in *Spred1-/-* mice. Regulation of the actin cytoskeleton is essential for formation and maintenance of dendritic spines, the actin-rich structures that are the main sites of synaptic connection. Stability of the cytoskeleton is critical for maintaining long term synaptic changes [76]. Additionally, many genes associated with ASD encode proteins that are involved in regulation of the actin cytoskeleton [77], and dysregulation of the actin cytoskeleton is seen in *Shank3* mouse models for syndromic ASD [78]. An earlier study in *Spred1-/-* mice saw deficits in hippocampal synaptic plasticity, but no changes in the total number of dendritic spines in dentate gyrus granule cells of the hippocampus [32]. However, this does not exclude the possibility of effects in other brain regions, or in spine maturation and spine dynamics, so further studies are warranted.

Although cognitive symptoms and ASD are a major factor negatively impacting quality of life for RASopathy patients, current treatment options for improving cognitive and social functioning in NF1 and Legius syndrome patients are insufficient. A primary target has been reduction of the overactivation of RAS-MAPK signaling. Preclinical work identifying that cholesterol-lowering statin drugs could improve cognition in *Nf1* mouse models by modulation of Ras isoprenylation [53] led to a number of clinical trials, but none have shown significant cognitive benefits of statins in patients [79–82]. An early stage clinical trial specifically looking at statin treatment in NF1 children with ASD demonstrated safety, but was not powered to detect behavioral alterations [83]. Ritalin is in use for treating ADHD symptoms in NF1 children, and has shown benefits for cognitive and attentional measures [84]. Following the identification of the HCN1 potassium channel as a critical interactor of NF1, the HCN channel agonist Lamotrigine was shown to rescue cognitive phenotypes in *Nf1+/−* mice [85]. Lamotrigine is currently being tested in a Phase 2 clinical trial for improving cognition in NF1 patients (NCT02256124). Recent work comparing the ASD profile of RASopathies saw that there were no significant differences between NF1, Noonan syndrome and CFC syndrome, suggesting that insights from one RASopathy may be generalized to other RASopathies [4]. This is further evidence for a role for RAS-MAPK signaling in ASD, which is supported by our data with MEK inhibition in this study.

MEK inhibitors are being investigated in the clinic for treating symptomatic and inoperable plexiform neurofibromas in NF1 patients, with PD325901 treatment demonstrating neurofibroma shrinkage in Phase 2 trials in adolescents and adults [56], and a Phase 2b trial currently following up this result in children and adults (NCT03962543). Recently a different MEK inhibitor, selumetinib, demonstrated tumor shrinkage and clinical benefit for children with plexiform neurofibromas shrinkage [86], and has now received FDA and EMA approval for use in pediatric NF1 patients. Notably, cognitive and behavioral assessments of patients enrolled in the selumetinib trial are now being followed as secondary outcome measures [87]. Given our findings demonstrating that PD325901 modulates social behavior in mouse models, this strongly suggests that ongoing and future clinical trials for PD325901 should also monitor cognitive and behavioral parameters as secondary outcomes.

## Limitations

Our study has several limitations. As this study was proof of concept that MEK inhibition could modulate social behavior in a RASopathy model, we limited our dose to 5 mg/kg. This dose was already demonstrated in mice to rapidly attenuate ERK phosphorylation [48, 49], and to successfully treat other RASopathy indications in mouse models, such as shrinking plexiform neurofibromas [50] and reversing growth defects [51]. The study where PD325901 shrank mouse plexiform neurofibromas in a mouse model saw effective tumor shrinkage both at the dose used in our study, 5 mg/kg, and at a lower dose, 1.5 mg/kg, and these doses were well-tolerated with no toxicity [50]. However, behavior was not examined in that study. Recent and ongoing clinical trials using PD325901 to treat plexiform neurofibromas have used a maximum daily dose of 8 mg/m^2^ [88], which roughly equates to 1.33 mg/kg in the mouse. However, this treatment course has been optimized for treating tumors, and it is not unreasonable to expect that a treatment course that was optimized to modulate behavior would be different. Therefore, we urge caution with direct comparisons between our studies and ongoing clinical trials. Additionally, as part of our proof of concept we employed an acute treatment paradigm, as a number of studies have demonstrated that cognitive phenotypes in RASopathy models can be reversed with acute treatment paradigms starting 3 days prior to testing. Lovastatin administered on such an acute regimen improved spatial memory impairments in both *Nf1+/−* mice and Noonan syndrome *Ptpn11*^*D62G/*+^ mice [52, 53]. Therefore, before making further translations to the clinic, future follow-up preclinical studies would be required to determine the minimum effective dose required to modulate social behavior in mouse models. This will need to include time course studies in order to characterize the effects of longer-term treatments with this drug on behavior.

Our study phenotyped *Spred1-/-* mice across a panel of behavior tests linked to social functioning, with the aim of identifying robust, reproducible phenotypes that could be used for screening drugs that modulate behavior in RASopathy models. This included testing mice for sociability and social preference, aggression and social dominance. However, there are other domains of social functioning in mice that are not captured by these tests, such as the social transfer of information [89], or emotional discrimination [90]. It will be of interest to investigate these other social domains in future studies to better characterize the social impairments of the *Spred1-/-* mouse model. Finally, Legius syndrome is caused by heterozygous mutations in *SPRED1* [16], whereas we have used a homozygous *Spred1* mouse model in order to be able to robustly detect phenotypes. The possibility that lower doses of the MEK inhibitor may be sufficient to have an effect in heterozygous patients should be taken into consideration.

## Conclusions

In conclusion, social behavior phenotypes can be modelled robustly in *Spred1-/-* mice, indicating its validity as a model for studying ASD in RASopathies. Social behavioral abnormalities in *Spred1-/-* mice are reversible upon acute pharmacological MEK inhibition, indicating a critical role for Ras-MAPK signaling in the regulation of social behavior and a novel therapeutic approach for Legius syndrome.

## Supplementary Information

**Additional file 1**. Supplementary Material.

## Data Availability

The datasets used and/or analyzed during the current study are available from the corresponding author on reasonable request.

## Abbreviations

ANOVA: Analysis of variance
ADHD: Attention deficit hyperactivity disorder
ASD: Autism spectrum disorder
CFC: Cardiofaciocutaneous syndrome
DMSO: Dimethyl sulfoxide
ERK: Extracellular signal-regulated kinases
EVH1: Enabled/VASP homology 1 domain
HCN1: Potassium/sodium hyperpolarization-activated cyclic nucleotide-gated channel 1
LTP: Long term potentiation
MEK: Mitogen-activated protein kinase
mTOR: Mammalian target of rapamycin
NF1: Neurofibromatosis type 1
Ras-MAPK: Ras-mitogen-activation protein kinase
ROCK: Rho-associated protein kinase
RRBs: Restrictive and repetitive behaviors
SPRED1: Sprouty related EVH1 domain containing 1
USV: Ultrasonic vocalization
WT: Wildtype
