# Supplementary material for: MEK inhibition ameliorates social behavior phenotypes in a Spred1 knockout mouse model for RASopathy disorders
Source: Mol Autism. 2021 Jul 26;12:53. doi: 10.1186/s13229-021-00458-2 (PMC8314535; doi:10.1186/s13229-021-00458-2)
Supplement: Supplementary file 1 — Additional file 1. Supplementary Material. [file 13229_2021_458_MOESM1_ESM.docx]

**MEK inhibition ameliorates social behavior phenotypes in a Spred1 knockout mouse model for RASopathy disorders**

**Borrie et. al.**

***Supplementary Material***

Supplementary Table 1

Supplementary Figures 1-6

Supplementary references

**Supplementary Table 1: Summary of Clinical Features Observed in Individuals Carrying a *SPRED1* Mutation, as Reported in Literature**

| Report | Brems et al. | Pasmant et al. | Spurlock et al. | Messiaen et al. | Muram-Zborovski et al. | Denayer et al. | Laycock-van Spyk et al. | Spencer et al. | Pasmant et al. | Benelli et al. | Sakai et al. | Hirata et al. | Bianchi et al. | Kimura et al. | Sekelska et al. | Bulteel et al. | Schluth-Bolard et al. | Pacot et al. | Witkowski et al, | Castellanos et al. | Bianchessi et al. | Bixel et al. | Orlandi et al. |
| --- | --- | --- | --- | --- | --- | --- | --- | --- | --- | --- | --- | --- | --- | --- | --- | --- | --- | --- | --- | --- | --- | --- | --- |
| Year | 2007 | 2009 | 2009 | 2009 | 2010 | 2011 | 2011 | 2011 | 2015 | 2015 | 2015 | 2015 | 2015 | 2017 | 2017 | 2018 | 2019 | 2019 | 2020 | 2020 | 2020 | 2020 | 2021 |
| *Ref.* | (1,2) | (3) | (4) | (5) | (6) | (7) | (8) | (9) | (10) | (11) | (12) | (13) | (14) | (15) | (16) | (17) | (18) | (19) | (20) | (21) | (22) | (23) | (24) |
| Number of individuals | 42 | 18 | 12 | 40 | 2 | 30 | 10 | 7 | 10 | 4 | 2 | 21 | 3 | 1 | 1 | 1 | 1 | 1 | 2 | 6 | 1 | 3 | 1 |
| Notes | *a* |  |  |  |  |  |  |  |  |  |  | *b* |  |  |  |  |  |  |  |  |  |  |  |
| ***Dermatological and pigmentation-related:*** |  |  |  |  |  |  |  |  |  |  |  |  |  |  |  |  |  |  |  |  |  |  |  |
| CALMs | 40/40 | 18 | 12 | 35 | 2 | 28 | 6 | 7 | 10 | 4 | 2 | 20 | 3 | 1 | 1 | 1 | 1 | 1 | 2 | 5 | 1 | 3 | 1 |
| Freckling | 13/39 | 14 | 7/11 | 17 | 2 | 10/27 | 2 | 2 |  | 1 | 2 | 10 | 3 | 1 |  |  |  | 1 | 2 |  | 1 | 3 |  |
| Lentigines |  |  |  |  |  |  |  |  | 2 |  |  |  |  |  |  |  |  |  |  |  |  |  |  |
| Depigmented macules/ Nevus Anemicus | 3 |  |  | 1 |  |  |  |  |  |  |  |  |  |  |  | 1 |  |  |  |  |  |  |  |
| Lisch nodules |  |  |  |  |  |  |  |  |  |  |  |  |  |  |  |  |  |  |  |  |  | 2 |  |
| ***Cognitive, Behavioral, Neurological*** |  |  |  |  |  |  |  |  |  |  |  |  |  |  |  |  |  |  |  |  |  |  |  |
| Learning disabilities | 8 | 4 | 0 | 1/38 | 2 | 14/25 |  | 1 | 2 | 1 |  | 1 |  |  | 1 |  |  |  | 1 |  |  |  |  |
| Intellectual disability |  |  |  |  |  |  | 1 |  |  |  |  |  |  |  |  |  | 1 |  | 1 |  |  |  |  |
| Developmental delay | 1 | 1 | 0 | 6/38 |  | 6/18 | 7 | 1 | 1 |  |  | 2 | 2 |  |  |  |  |  |  |  |  |  |  |
| ADHD, ADD, or hyperactivity | 4 | 1 |  | 5/38 |  | 5/14 |  | 2 |  |  |  |  |  |  |  |  |  |  |  |  |  |  |  |
| ASD, autistic features, or PDD-NOS | 3 |  |  |  |  | 1/14 | 1 |  |  |  |  |  |  |  |  |  |  |  |  |  |  |  |  |
| Headaches |  | 4 |  |  |  | 2 |  |  |  |  |  |  |  |  |  |  |  |  |  |  |  |  |  |
| Hearing loss |  |  |  | 3 |  | 1 |  |  |  |  |  |  |  |  |  |  |  |  |  |  |  |  |  |
| Seizures |  | 1 |  | 2 |  | 1 | 1 |  |  | 1 |  |  |  |  |  |  |  |  |  |  |  |  |  |
| ***Skeletal:*** |  |  |  |  |  |  |  |  |  |  |  |  |  |  |  |  |  |  |  |  |  |  |  |
| Relative or absolute macrocephaly | 13/31 | 1 | 1/8 | 7/31 | 1 | 3/24 | 3 |  |  | 1 |  | 3 | 0 |  |  |  | 1 |  |  |  | 1 | 1 |  |
| Short stature (<5th percentile) | 3/30 |  |  | 1/30 | 0 | 6/26 |  | 1 |  |  |  | 1 |  |  |  |  |  |  | 1 |  |  |  |  |
| Noonan-like face or characteristics, hypertelorism | 5 |  | 0 | 1 |  | 12 |  | 1 |  |  |  | 1 | 0 |  |  |  | 1 |  | 1 | 1 |  |  |  |
| Pectus excavatum/ carinatum | 3 |  |  | 3 |  | 7 | 1 | 1 |  |  |  |  |  |  |  |  |  |  |  |  |  |  |  |
| Scoliosis |  |  |  |  |  | 4 | 1 |  |  |  |  | 3 |  |  |  |  |  |  |  |  |  |  |  |
| Polydactyly |  |  |  | 1 |  | 2 |  |  |  |  |  |  |  |  |  |  |  |  |  |  |  |  |  |
| Clinodactyly 5th finger |  |  |  | 2 |  |  |  |  |  |  |  |  |  |  |  |  |  |  |  |  |  |  |  |
| ***Tumoral:*** |  |  |  |  |  |  |  |  |  |  |  |  |  |  |  |  |  |  |  |  |  |  |  |
| Tumors | 3 | 1 |  | 3 |  | 2 |  |  |  |  |  |  |  |  |  |  |  |  |  |  |  |  |  |
| Lipomas | 14 | 2 |  | 2 |  | 2/15 |  | 1 |  |  |  |  |  |  |  |  |  |  |  |  |  |  |  |
| ***Vascular:*** |  |  |  |  |  |  |  |  |  |  |  |  |  |  |  |  |  |  |  |  |  |  |  |
| Vascular malformation | 1 |  | 1 | 2 |  |  |  |  |  |  |  |  |  |  |  |  |  |  |  |  |  |  |  |
| Valve problems | 1 |  |  | 1 |  | 1 |  |  |  |  |  |  |  |  |  |  |  |  | 1 |  |  |  |  |

Notes:

a) UAB31, 43, 48 are not included here as they are included in the clinical cohort of Ref. (5). For UZL2 III4, UZL2 III8 and UZL2 III9, additional clinical data was reported in Ref. (2). and both sources are summarized here. Two additional patients from Ref. (2), UZ Brussel, Belgium 1 and NIH, USA IV3, had cognitive/behavioural clinical phenotype data reported in that study but without other clinical information, and these patients have been include in this column as well.

b) This column summarizes data from the new patients reported in this study (13) and from 4 patients initially reported in Ref. (25) that did not have clinical data reported previously.

CALM – café au lait macule; ADHD - Attention deficit hyperactivity disorder; ADD - Attention deficit disorder; ASD – autism spectrum disorder; PDD-NOS - Pervasive Developmental Disorder-Not Otherwise Specified.

**
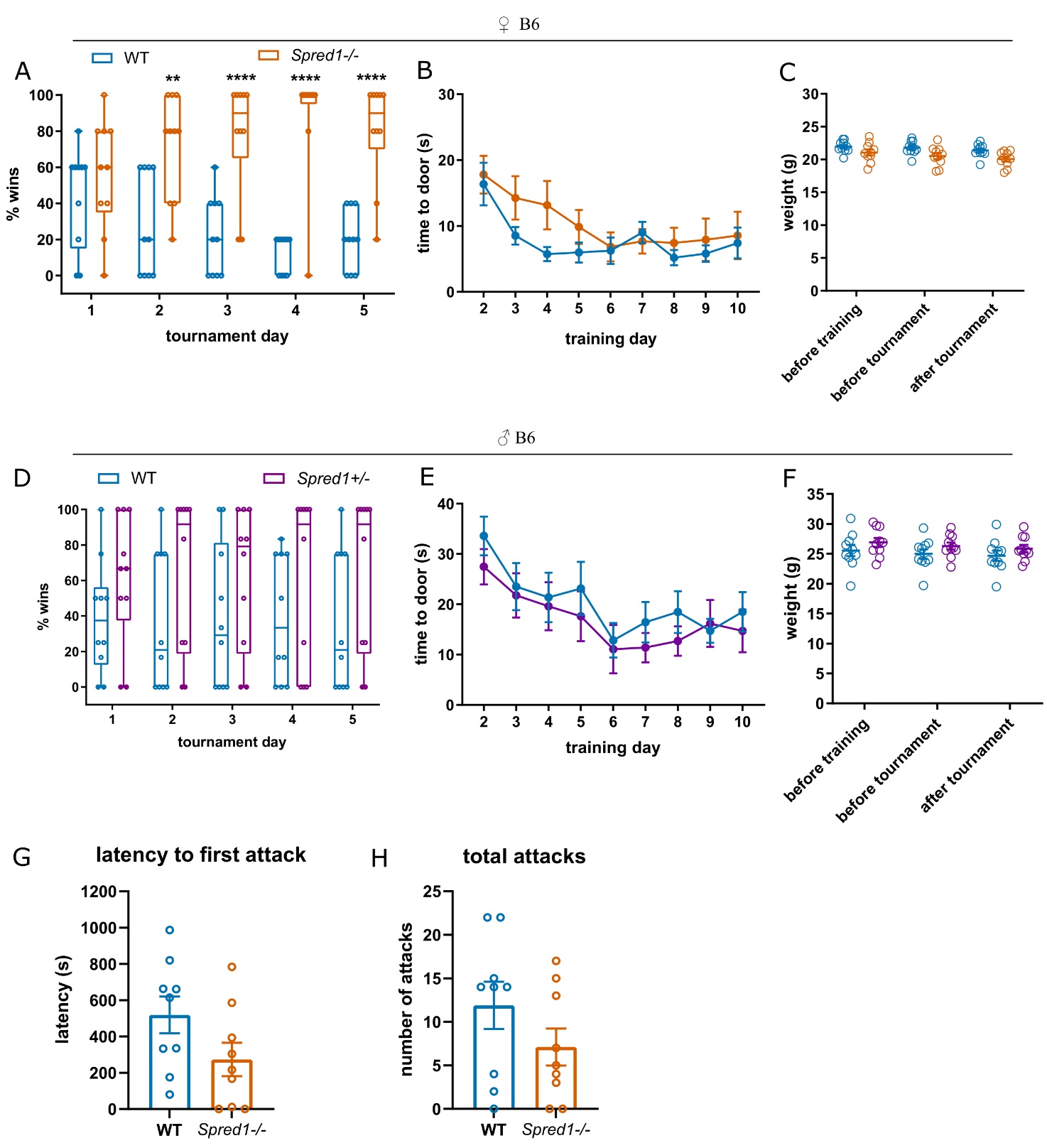
**

**Supplementary Figure 1. Social dominance and aggression tests in *Spred1-/-* and *Spred1+/-* mice on the B6 background**

**A-C)** Automated tube test for social dominance in female *Spred1-/-* mice on the B6 background. **A)** Percentage of matches won per day in the tube test across 5 days of tournaments between WT and *Spred1-/-* female mice on the B6 background. Significant effect of genotype on days 2-5 (Two tailed binomial test compared to chance (50%): day 2 *p* = 0.0013; day 3 *p* < 0.0001; day 4 *p* < 0.0001; day 5 *p* < 0.0001). Box and whisker plots of median and quartiles, n = 10 mice/genotype. **B)** Tube test training data from female WT and *Spred1-/-* mice in the B6 background, time to center door (s) (2 Way ANOVA with repeated measures: effect of day F(8,64) = 3.296, *p* = 0.0033; no effect of genotype, no interaction. mean ± SEM). **C)** Weight (g) before and after tube tournaments for WT and *Spred1-/-* female mice on the B6 background. B) and C) are mean ± SEM.

**D-F)** Automated tube test for social dominance in male *Spred1+/-* mice on the B6 background. **D)** Mean percentage of matches won per day in the tube test across 5 days of tournaments between WT and *Spred1+/-* mice. (Two tailed binomial test compared to chance (50%), days 1-5 p > 0.1). **E)** Tube test training data from male WT and *Spred1-/-* mice in the C57BL6J background, time to center door (s). 2 Way ANOVA: effect of day F(8,144) = 14.92, *p* < 0.0001; no effect of genotype, no interaction. **F)** Weight (g) before and after tube tournaments for WT and *Spred1+/-* male mice on the B6 background. n = 10 mice/genotype. E-F) are mean ± SEM.

**G-H)** Resident intruder test in *Spred1-/-* male mice and WT controls. **G)** Latency to first attack in seconds, unpaired t-test, t=1.794, df=16, *p =* 0.0918. **H)** Total attacks in 30 minutes, unpaired t-test, t=1.386, df=16, *p =* 0.1848. n= 9 mice/genotype, mean ± SEM.


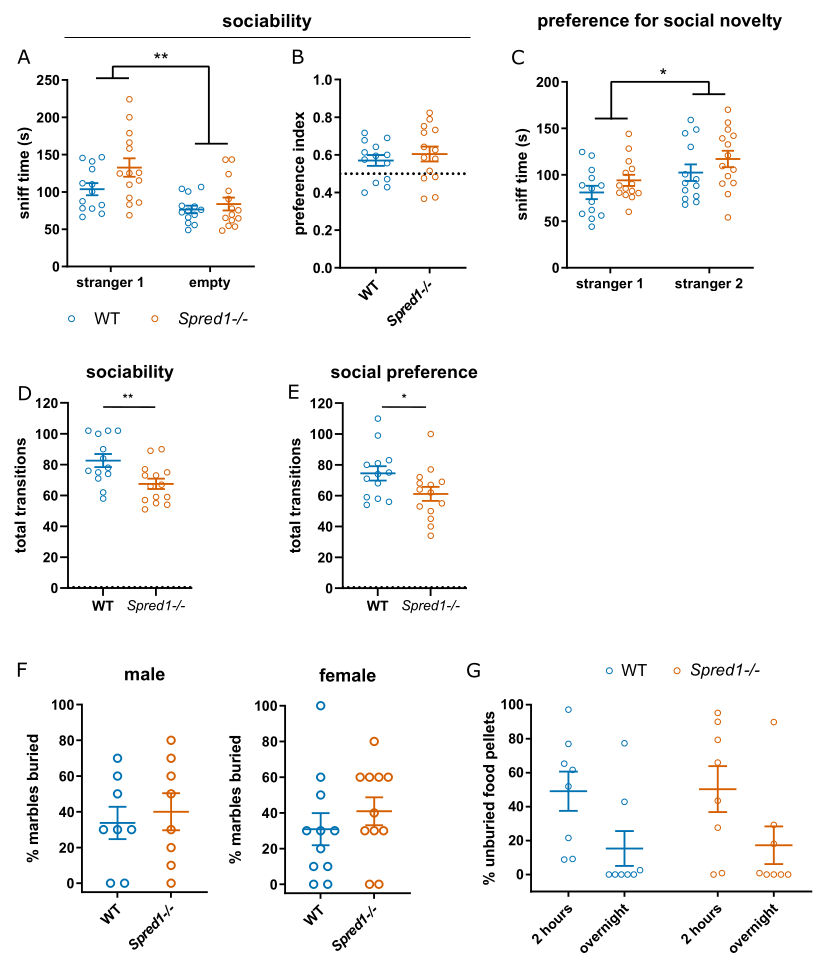


**Supplementary Figure 2. Unaltered social behavior in the three-chamber test and normal digging behavior in *Spred1-/-* mice**

**A-E)** Three chamber sociability test in female *Spred1-/-* and WT mice on the B6 background. **A)** Both genotypes spent more time interacting with stranger 1 compared to an empty cup, assessed by time spent sniffing (2-way ANOVA, effect of chamber side (stranger 1 vs empty): F(1,25) = 11.06, ***p* = 0.0027; effect of genotype: F(1,25) = 9.258, ***p* = 0.0054). **B)** Despite the genotype effect seen in A), when a social preference index was calculated for stranger 1 vs empty, no genotype difference was seen in preference (Unpaired t-test, t = 0.6898, df = 25, *p* = 0.4967). **C)** Social preference for a novel stranger (stranger 2) over the original stranger 1 was observed in both genotypes (2-way ANOVA, effect of chamber side (stranger 1 vs stranger 2): F(1,25) = 6.396, **p* = 0.0181; no effect of genotype). **D)** Total transitions between chambers for the sociability phase. Unpaired t-test, t=2.805, df=25, *p =* 0.0096. **E)** Total transitions between chambers for the social preference phase. Unpaired t-test, t=2.061, df=25, *p =* 0.0499. n = 13-14 mice/genotype, data presented as mean ± SEM. **F)** Percentage of marbles buried in the marble burying test in male and female WT and *Spred1-/-* mice (Males: unpaired t-test, t = 0.4545, df = 14, *p* = 0.6564. Females: unpaired t-test, t = 0.8402, df = 20, *p* = 0.4107). Males n = 8 mice per genotype, females n = 11 mice per genotype. **G)** Burrowing behavior in female mice as measured by the percentage of food pellets (by weight) unburied after 2 hours and after overnight. RM ANOVA, F < 1.00; p > 0.1. n = 8 mice/genotype. All data are mean±SEM.


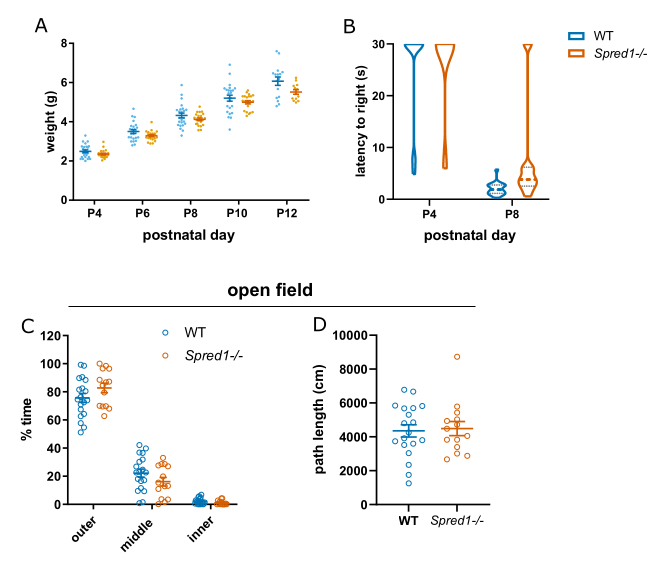


**Supplementary Figure 3. Neonatal body weight and righting reflex, and adult open field exploration in *Spred1-/-* mice**

**A)** Body weight of pups over time (2-way ANOVA with repeated measures: main effect of age, F(4,159) = 1325, *****p* < 0.0001, no effect of genotype, no interaction). Mean ± SEM, n = 22- 24 mice. **B)** Righting reflex of pups on P4 and P8, measured as the latency to right (2-way ANOVA with repeated measures: main effect of age, F(1,29) = 77.15, *p* < 0.0001; no effect of genotype, no interaction). n = 12-17 mice/genotype. Violin plots show median and quartiles.

**C-D)** Open field in adult *Spred1-/-* mice and WT controls (male and female mice pooled, n = 14-16 mice/genotype). C) Percentage of time spent in the three zones of the circular open field apparatus. D) Total pathlength in the open field. Data are presented as mean±SEM.


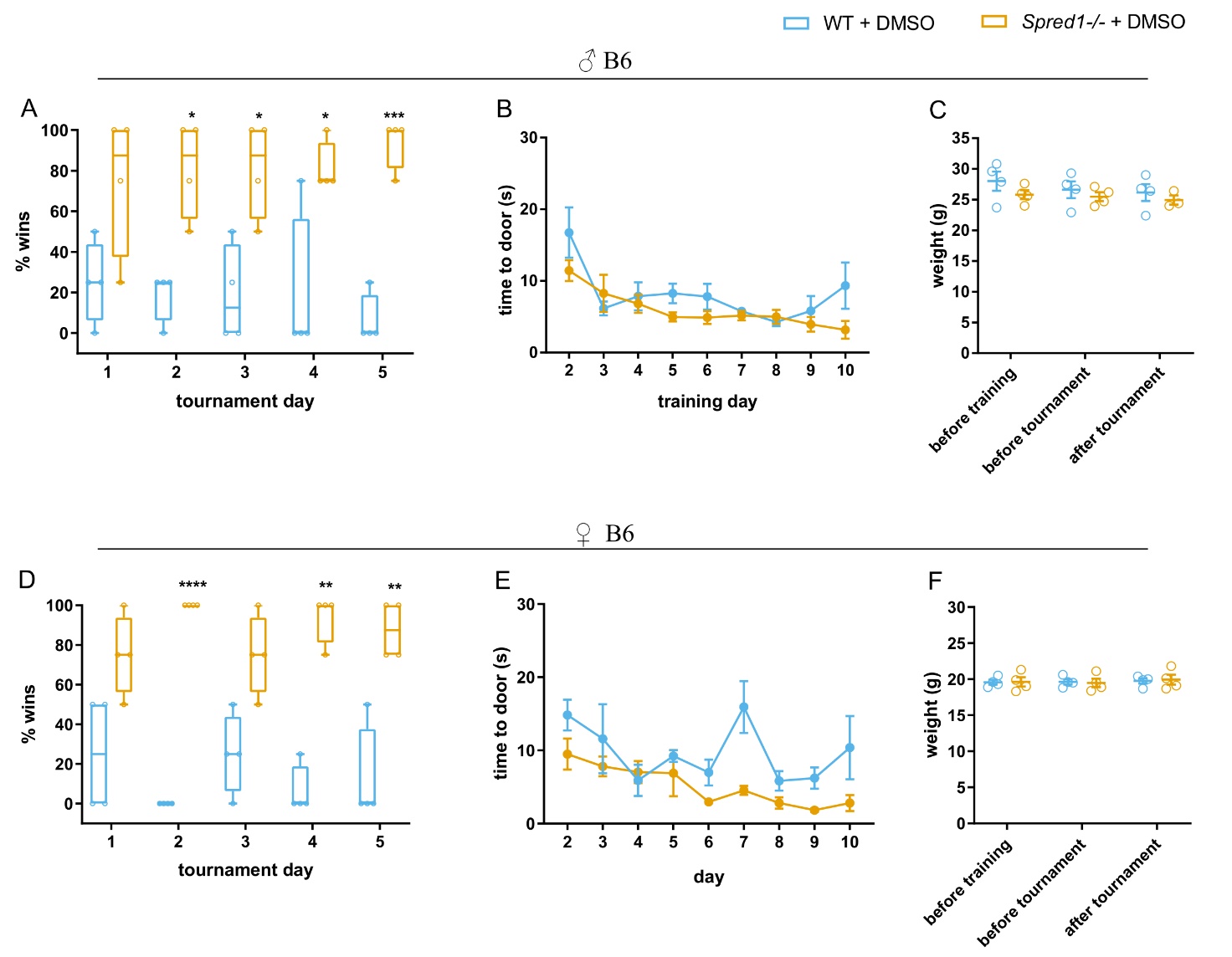


**Supplementary Figure 4. Automated tube test in vehicle treated *Spred1-/-* and WT mice on the B6 background**

Male (A-C) and female (D-F) cohorts. **A)** Mean percentage of matches won per day in the tube test across 5 days of tournaments between male WT mice + DMSO vehicle and *Spred1-/-* mice + DMSO vehicle, significant differences day 2-5. Two tailed binomial test compared to chance (50%): significant differences day 2-5. Day 2 *p* = 0.0213; day 3 *p* = 0.0213; day 4 *p* = 0.0213; day 5 *p* = 0.0005. n = 4 mice/genotype. **B)** Time (s) to reach the center door of the automated tube during days 2-10 of tube test training. **C)** Body weight (g) before treatment, before tournaments, and after tournaments. **D)** Mean percentage of matches won per day in the tube test across 5 days of tournaments between female WT mice + DMSO vehicle and *Spred1-/-* mice + DMSO vehicle. Two tailed binomial test compared to chance (50%): significant differences day 2-5. Day 2 *p* < 0.0001; Day 4 *p* = 0.005; Day 4 *p* = 0.0042. n = 4 mice/genotype. **E)** Time (s) to reach the center door of the automated tube during days 2 – 10 of tube test training. **F)** Body weight (g) before treatment, before tournaments, and after tournaments. Box and whisker plots show of median and quartiles; all other graphs mean ± SEM. * p < 0.05, ** p < 0.01, *** p < 0.001, **** p < 0.0001.


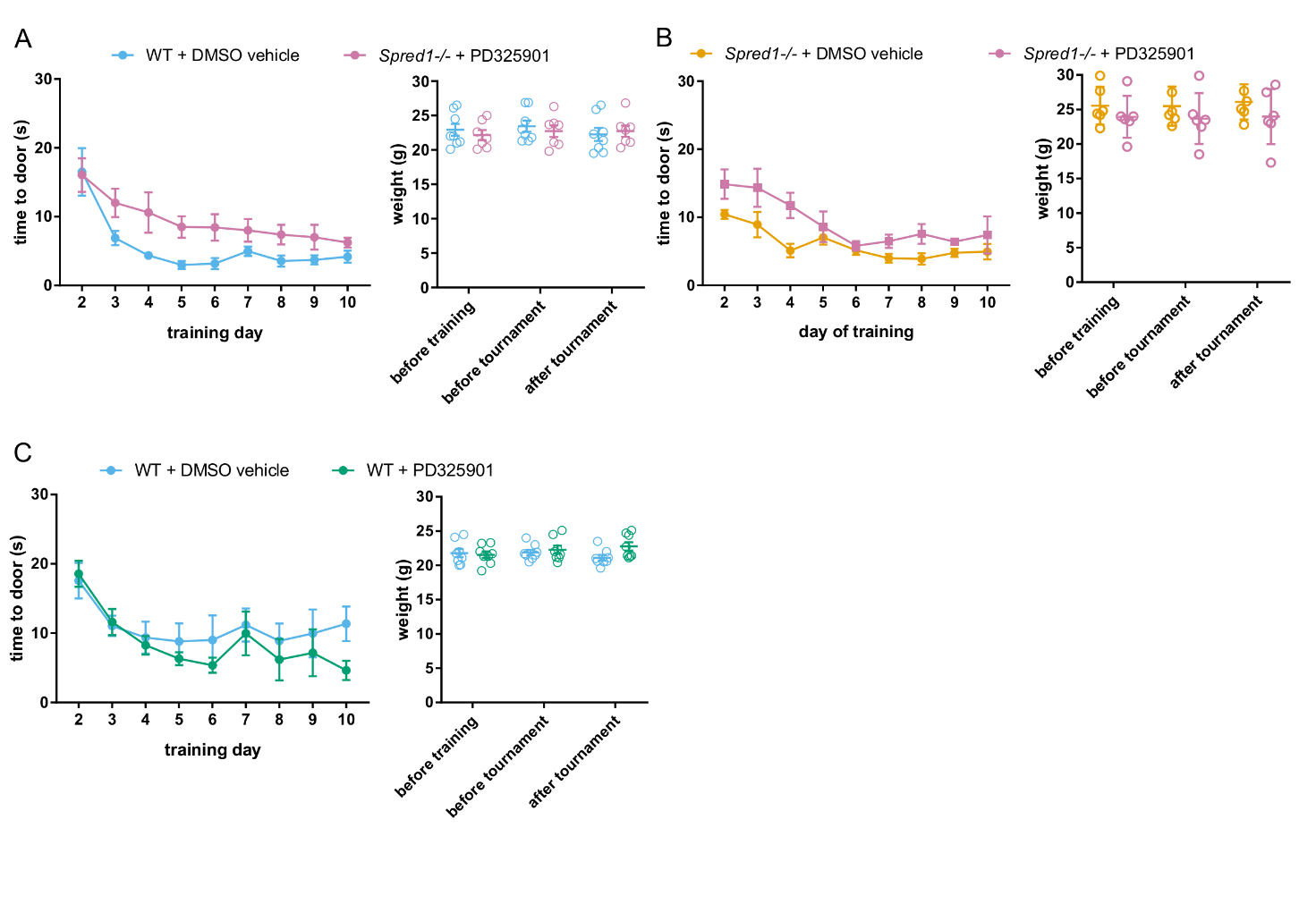


**Supplementary Figure 5. Automated tube test training data and weight data from PD325901 and DMSO treated cohorts.**

**A)** Training data and weight data for WT + DMSO v Spred1-/- + PD325901 cohort. Left, time (s) to reach the center door of the automated tube during training. Right, body weight (g) before treatment, before tournaments, and after tournaments. n = 8 mice/group. **B)** Training data and weight data for *Spred1-/-* + DMSO v *Spred1-/-* + PD325901 cohort. Left, time (s) to reach the center door of the automated tube during training. Right, body weight (g) before treatment, before tournaments, and after tournaments. n = 6 mice/group. **C)** Training data and weight data for WT + DMSO v WT + PD325901 cohort. Left, time (s) to reach the center door of the automated tube during training. Right, body weight (g) before treatment, before tournaments, and after tournaments. n = 8 mice/group. Mean ± SEM. All mice were on the B6 background.


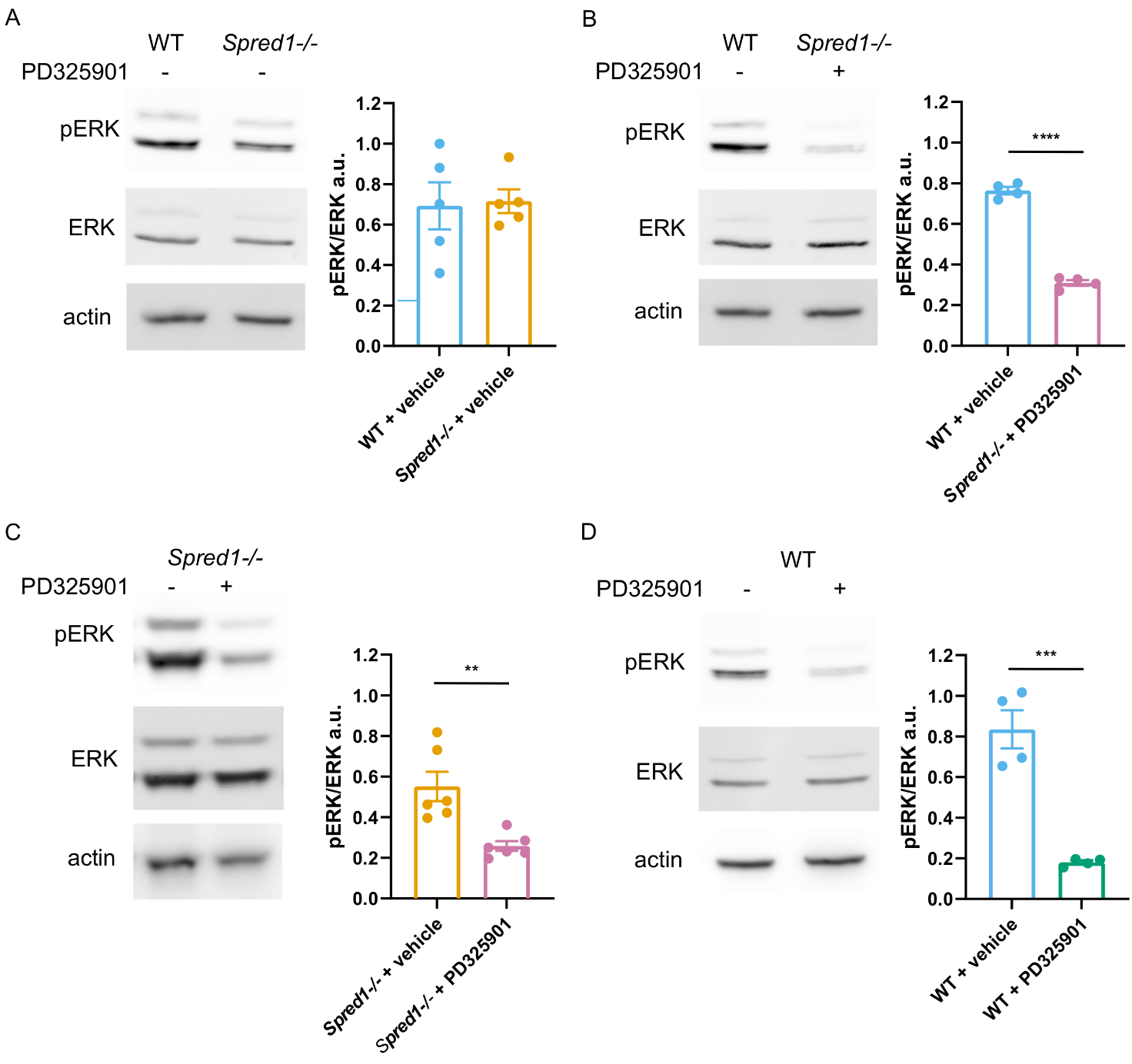


**Supplementary Figure 6. Hippocampal ERK activation is attenuated by PD325901 treatment.**

**A)** Western blot of hippocampal lysates and quantification from WT + vehicle and *Spred1-/-* + vehicle cohort, showing no genotype effect on pERK levels (unpaired t-test, t = 0.1777, df = 8, *p* = 0.8634). **B)** Western blot of hippocampal lysates and quantification from a WT + vehicle and *Spred1-/-* + PD325901 cohort demonstrating significant attenuation of pERK/ERK levels in *Spred1-/-* + PD325901 hippocampi (unpaired t-test, t = 19.69, df = 6, *p* < 0.0001). **C)** Western blot of hippocampal lysates and quantification from a *Spred1-/-* + vehicle and *Spred1-/-* + PD325901 cohort, demonstrating significant attenuation of pERK/ERK levels in *Spred1-/-* + PD325901 hippocampi (unpaired t-test, t = 3.846, df = 10, *p* = 0.0032). **D)** Western blot of hippocampal lysates and quantification from a WT + vehicle and WT + PD325901 cohort demonstrating significant attenuation of pERK/ERK levels in WT + PD325901 hippocampi (unpaired t-test, t = 6.957, df = 6, *p* = 0.0004). All groups were n = 4 mice per genotype, except for C), n = 6/genotype. Mean ± SEM.

**Supplementary references**

1. Brems H, Chmara M, Sahbatou M, Denayer E, Taniguchi K, Kato R, *et al.* (2007): Germline loss-of-function mutations in SPRED1 cause a neurofibromatosis 1-like phenotype. *Nature genetics* 39: 1120–6.

2. Denayer E, Descheemaeker MJ, Stewart DR, Keymolen K, Plasschaert E, Ruppert SL, *et al.* (2011): Observations on intelligence and behavior in 15 patients with Legius syndrome. *American Journal of Medical Genetics, Part C: Seminars in Medical Genetics* 157: 123–128.

3. Pasmant E, Ballerini P, Lapillonne H, Perot C, Vidaud D, Leverger G, Landman-Parker J (2009): *SPRED1 Disorder and Predisposition to Leukemia in Children*, vol. 114. https://doi.org/10.1182/blood-2009-04-218503

4. Spurlock G, Bennett E, Chuzhanova N, Thomas N, Jim H-P, Side L, *et al.* (2009): SPRED1 mutations (Legius syndrome): another clinically useful genotype for dissecting the neurofibromatosis type 1 phenotype. *Journal of Medical Genetics* 46: 431–437.

5. Messiaen L, Yao S, Brems H, Callens T, Sathienkijkanchai A, Denayer E, *et al.* (2009): Clinical and mutational spectrum of neurofibromatosis type 1-like syndrome. *JAMA* 302: 2111–8.

6. Muram-Zborovski TM, Stevenson DA, Viskochil DH, Dries DC, Wilson AR, Mao R (2010): SPRED 1 Mutations in a Neurofibromatosis Clinic. *J Child Neurol* 25: 1203–1209.

7. Denayer E, Chmara M, Brems H, Kievit AM, Van Bever Y, Van Den Ouweland AMW, *et al.* (2011): Legius syndrome in fourteen families. *Human Mutation* 32: E1985–E1998.

8. Laycock-van Spyk S, Jim HP, Thomas L, Spurlock G, Fares L, Palmer‐Smith S, *et al.* (2011): Identification of five novel SPRED1 germline mutations in Legius syndrome. *Clinical Genetics* 80: 93–96.

9. Spencer E, Davis J, Mikhail F, Fu C, Vijzelaar R, Zackai EH, *et al.* (2011): Identification of SPRED1 deletions using RT-PCR, multiplex ligation-dependent probe amplification and quantitative PCR. *American Journal of Medical Genetics Part A* 155: 1352–1359.

10. Pasmant E, Gilbert-Dussardier B, Petit A, de Laval B, Luscan A, Gruber A, *et al.* (2015): SPRED1, a RAS MAPK pathway inhibitor that causes Legius syndrome, is a tumour suppressor downregulated in paediatric acute myeloblastic leukaemia. *Oncogene* 34: 631–638.

11. Benelli E, Bruno I, Belcaro C, Ventura A, Berti I (2015): Legius syndrome: case report and review of literature. *Italian Journal of Pediatrics* 41: 8.

12. Sakai N, Maeda T, Kawakami H, Uchiyama M, Harada K, Tsuboi R, Mitsuhashi Y (2015): Family with Legius syndrome (neurofibromatosis type 1-like syndrome). *The Journal of Dermatology* 42: 703–705.

13. Hirata Y, Brems H, Suzuki M, Kanamori M, Okada M, Morita R, *et al.* (2015): Interaction between a domain of a negative regulator of the RAS-ERK pathway, SPRED1, and the GTPase-Activating Protein-Related Domain of neurofibromin is implicated in Legius Syndrome and Neurofibromatosis Type 1. *Journal of Biological Chemistry* 291: jbc.M115.703710.

14. Bianchi M, Saletti V, Micheli R, Esposito S, Molinaro A, Gagliardi S, *et al.* (2015): Legius Syndrome: two novel mutations in the SPRED1 gene [no. 1]. *Human Genome Variation* 2: 1–3.

15. Kimura R, Yoshida Y, Maruoka R, Kosaki K, Yamamoto O (2017): Legius syndrome: A case report. *The Journal of Dermatology* 44: 459–460.

16. Sekelska M, Briatkova L, Olcak T, Bolcekova A, Ilencikova D, Kadasi L, Zatkova A (2017): The first Slovak Legius syndrome patient carrying the SPRED1 gene mutation. *Gen Physiol Biophys* 36: 205–210.

17. Bulteel C, Morren M-A, De Haes P, Denayer E, Legius E, Brems H (2018): Nevus anemicus and RASopathies. *JAAD Case Rep* 4: 390–391.

18. Schluth-Bolard C, Diguet F, Chatron N, Rollat-Farnier P-A, Bardel C, Afenjar A, *et al.* (2019): Whole genome paired-end sequencing elucidates functional and phenotypic consequences of balanced chromosomal rearrangement in patients with developmental disorders. *Journal of Medical Genetics* 56: 526–535.

19. Pacot L, Burin des Roziers C, Laurendeau I, Briand-Suleau A, Coustier A, Mayard T, *et al.* (2019): One NF1 Mutation may Conceal Another [no. 9]. *Genes* 10: 633.

20. Witkowski L, Dillon MW, Murphy E, Lebo MS, Mason‐Suares H (2020): Expanding the Noonan spectrum/RASopathy NGS panel: Benefits of adding NF1 and SPRED1. *Molecular Genetics & Genomic Medicine* 8: e1180.

21. Castellanos E, Rosas I, Negro A, Gel B, Alibés A, Baena N, *et al.* (2020): Mutational spectrum by phenotype: panel-based NGS testing of patients with clinical suspicion of RASopathy and children with multiple café-au-lait macules. *Clinical Genetics* 97: 264–275.

22. Bianchessi D, Ibba MC, Saletti V, Blasa S, Langella T, Paterra R, *et al.* (2020): Simultaneous Detection of NF1, SPRED1, LZTR1, and NF2 Gene Mutations by Targeted NGS in an Italian Cohort of Suspected NF1 Patients [no. 6]. *Genes* 11: 671.

23. Bixel KD, Cano MJ, Johnson DM, Gomez B, Lobsinger LV, Valentin FE, *et al.* (2020): Lisch nodules and iris mammillations in two siblings with familial legius syndrome. *Clinical Case Reports* 8: 1867–1871.

24. Orlandi V, Cavarzere P, Palma L, Gaudino R, Antoniazzi F (2021): Central precocious puberty in a girl with LEGIUS syndrome: an accidental association? *Italian Journal of Pediatrics* 47: 50.

25. Brems H, Pasmant E, Van Minkelen R, Wimmer K, Upadhyaya M, Legius E, Messiaen L (2012): Review and update of SPRED1 mutations causing legius syndrome. *Human Mutation* 33: 1538–1546.
